# Analyses of the radiation of birnaviruses from diverse host phyla and of their evolutionary affinities with other double-stranded RNA and positive strand RNA viruses using robust structure-based multiple sequence alignments and advanced phylogenetic methods

**DOI:** 10.1186/1471-2148-13-154

**Published:** 2013-07-17

**Authors:** Jean-François Gibrat, Mahendra Mariadassou, Pierre Boudinot, Bernard Delmas

**Affiliations:** 1 INRA, UR 1077 Mathématique, Informatique et Génome 78350, Jouy-en-Josas, France; 2 INRA, UR 892 Virologie et Immunologie Moléculaires 78350, Jouy-en-Josas, France

**Keywords:** RNA-dependent RNA polymerase, Capsid protein, Double-stranded RNA viruses, Positive strand RNA viruses, Rotifer, Maximum likelihood phylogeny method, Bayesian phylogeny method, Structure-based alignments

## Abstract

**Background:**

Birnaviruses form a distinct family of double-stranded RNA viruses infecting animals as different as vertebrates, mollusks, insects and rotifers. With such a wide host range, they constitute a good model for studying the adaptation to the host. Additionally, several lines of evidence link birnaviruses to positive strand RNA viruses and suggest that phylogenetic analyses may provide clues about transition.

**Results:**

We characterized the genome of a birnavirus from the rotifer *Branchionus plicalitis*. We used X-ray structures of RNA-dependent RNA polymerases and capsid proteins to obtain multiple structure alignments that allowed us to obtain reliable multiple sequence alignments and we employed “advanced” phylogenetic methods to study the evolutionary relationships between some positive strand and double-stranded RNA viruses. We showed that the rotifer birnavirus genome exhibited an organization remarkably similar to other birnaviruses. As this host was phylogenetically very distant from the other known species targeted by birnaviruses, we revisited the evolutionary pathways within the *Birnaviridae* family using phylogenetic reconstruction methods. We also applied a number of phylogenetic approaches based on structurally conserved domains/regions of the capsid and RNA-dependent RNA polymerase proteins to study the evolutionary relationships between birnaviruses, other double-stranded RNA viruses and positive strand RNA viruses.

**Conclusions:**

We show that there is a good correlation between the phylogeny of the birnaviruses and that of their hosts at the phylum level using the RNA-dependent RNA polymerase (genomic segment B) on the one hand and a concatenation of the capsid protein, protease and ribonucleoprotein (genomic segment A) on the other hand. This correlation tends to vanish within phyla. The use of advanced phylogenetic methods and robust structure-based multiple sequence alignments allowed us to obtain a more accurate picture (in terms of probability of the tree topologies) of the evolutionary affinities between double-stranded RNA and positive strand RNA viruses. In particular, we were able to show that there exists a good statistical support for the claims that dsRNA viruses are not monophyletic and that viruses with permuted RdRps belong to a common evolution lineage as previously proposed by other groups. We also propose a tree topology with a good statistical support describing the evolutionary relationships between the *Picornaviridae*, *Caliciviridae*, *Flaviviridae* families and a group including the *Alphatetraviridae*, *Nodaviridae*, *Permutotretraviridae*, *Birnaviridae*, *and Cystoviridae* families.

## Background

Birnaviruses define a family of non-enveloped bi-segmented double-stranded RNA viruses. The birnavirus capsid is made of a unique icosahedral T = 13 shell composed of 260 trimers of viral protein 2 (VP2) [1]. Internal to the virion are VP3, which forms a ribonucleoprotein complex with the genomic RNA [2], and VP1, the viral RNA-dependent RNA polymerase (RdRp), which is found both free and covalently attached to the genomic RNA [3]. While the smaller segment B has an open reading frame (ORF) coding for VP1, the segment A encodes the polyprotein precursor pVP2-VP4-VP3. VP4 is a protease that cleaves its own N and C termini off the polyprotein, thus also releasing pVP2 (the precursor of the capsid protein, VP2) and VP3 within the infected cell [4]. Subsequent serial cleavages at the C-terminus of pVP2 upon particle assembly yield mature VP2 and several peptides that remain within the virion [5,6]. The crystal structures of subviral particles (made of 20 trimers of VP2) of two birnaviruses have been reported [1,7-9]. The tertiary structure of VP2 comprises three distinct domain termed base (B), shell (S) and projection (P). Both domains S and P are folded as jelly roll β-barrels, oriented tangentially and radially, respectively, with respect to the icosahedral particle.

**Figure 1:**
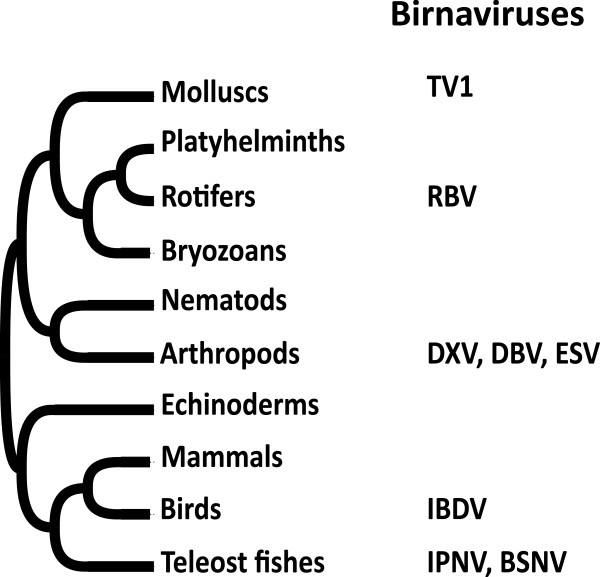
**Birnavirus host specificities in animal phyla.** Birnaviruses infects species belonging to two vertebrate phyla, birds and teleost fishes as well as to three invertebrate phyla, arthropods, mollusks ad rotifers. They are reported as TV1, Tellina virus 1; RBV, Rotifer birnavirus; DXV, Drosophila X virus; DBV, Drosophila B virus; ESV, Espirito Santo virus; IBDV, infectious bursal disease virus; IPNV, infectious pancreatic necrosis virus; BSNV, blotched snakehead virus.

The evolution of birnaviruses has to be considered in the context of their wide host range, from vertebrates to arthropods (ecdyzozoa), mollusks (lophotrochozoa) and rotifers (Figure 1). Six genetic clusters have been identified in the *Birnaviridae* family [10,11]. Three of them define viruses infecting a unique phylum, the vertebrates: the infectious bursal disease virus (IBDV) infects birds, while the blotched snakehead virus (BSNV), the infectious pancreatic virus (IPNV) and its allies infect fishes. The other clusters infect i) insects such as birnaviruses found in dipters, like the Drosophila X virus (DXV), the Drosophila B virus (DBV) and the Esperito Santo virus (ESV) which has been discovered in mosquito cells co-infected with dengue-2 virus [11] and ii) mollusks such as the Tellina virus 1 (TV-1) described from a bivalve [10]. Birnaviruses from rotifers have not been genetically characterized.

**Figure 2:**
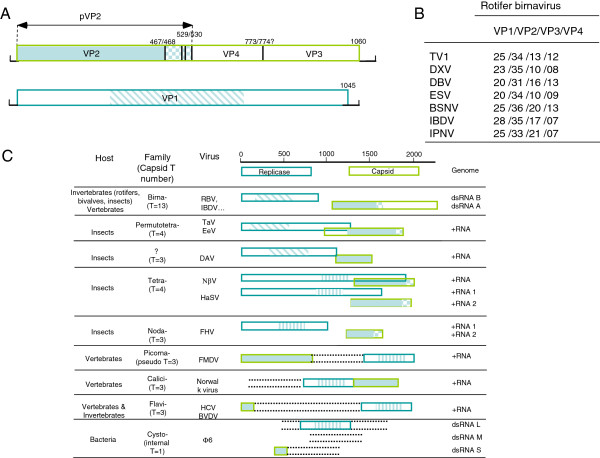
**Schematic representation of the genomic organization of different dsRNA and +sRNA viruses.** The polyprotein RBV ORF encompasses most of the genome segment A and contains the pVP2-VP4-VP3 sequence. The cleavage sites of the VP4 protease are indicated by vertical lines and their location is given by the P1/P1’ amino acid number. pVP2 is the VP2 capsid protein precursor while VP3 acts as a ribonucleoprotein that is associated to the genomic RNA. No additional open reading frame was evidenced in this segment in contrast to what is observed in other birnaviruses. The second genomic segment encodes VP1, the RNA-dependent RNA polymerase. B- Percentage of amino acid identity between the RBV proteins and their birnavirus homologs. C- Genetic organization of the double-stranded and positive strand RNA viruses used in this study. Shown are selected conserved domains of replicative and capsid proteins of birnaviruses (RBV, IBDV and others), TaV/EeV (Thosea asigna virus/Euprosterna elaeasa virus), DAV (Drosophila A virus), NβV (Nudaurelia β virus) and HaSV (Helicoverpa armigera stunt virus), FHV (Flock house virus), FMDV (Foot-and-mouth disease virus), Norwalk virus, HCV (Hepatitis C virus) and BVDV (bovine viral diarrhea virus), and phage Φ6. The conventional replicases are indicated with vertical hatched bars while permuted RdRp are represented with slanting bars.

Birnaviruses display unique features among double stranded RNA (dsRNA) viruses that are shared by positive single stranded (+ssRNA) RNA viruses such as nodaviruses [1,12] and alphatetraviruses (previously recognized as tetraviruses, [13]). The birnavirus VP2 capsid protein is structurally similar to the capsid protein of nodaviruses (capsid T = 3 triangulation number) or alphatetraviruses (T = 4). Also, a structural peptide that permeabilizes membranes and translocates the viral genome or the replication machinery into host cells - named γ peptide in nodaviruses and pep46 for IBDV- is found associated to the particle of both noda/alphatetra-viruses and birnaviruses. In both cases, the capsid protein and γ/pep46 peptide result from the cleavage of a capsid pre-protein at its C-terminus (Figure 2). Birnaviruses and noda/alphatetraviruses also share similar genomic arrangement and replication strategy. The bisegmented nature of the birnavirus genome, with one segment encoding the capsid protein and the second the RNA polymerase, is also retrieved in nodaviruses and in some alphatetraviruses. Importantly, the RdRp of two +ssRNA viruses (*Thosea asigna* virus and *Euprosterna elaeasa* virus) formerly related to the tetraviruses – and recently grouped in a separate family, the *Permutotetraviridae* (T = 4) [13] – shares with the birnavirus VP1 a unique polymerase motif rearrangement that has been found otherwise only in Drosophila A virus and the plant alpha-like virus Grapewine virus Q. The latter is likely to have evolved independently [14]. These polymerases have their catalytic motifs arranged in the permuted order C-B-A in the primary sequence of the palm domain, in contrast to the conventional A-B-C order found in all other viruses and organisms. However, in contrast to birnaviruses, the genome of the permutotetraviruses is mono-segmented with the capsid gene expressed through the synthesis of a subgenomic RNA and an additional capsid maturation processing occurring at the N-terminal domain of the pre-protein.

The crystal structures of the IBDV and IPNV RdRp show that it adopts an active site topology that was not previously observed in other polymerases [15,16].

The birnavirus Vpg-linked genome replication strategy, which has been retrieved in many +ssRNA viruses including picornaviruses and caliciviruses, is an additional common feature bringing birnaviruses and some other/additional +ssRNA viruses together.

Host specificity is also a property shared among birnaviruses and noda-, alphatetra- and permutotetraviruses. A growing number of birnaviruses are characterized in insects [11,17,18], which constitute the main host phylum of the three +ssRNA families (*Noda*-, *Alphatetra*- *and Permutotetraviridae*). Taken together, these observations strongly suggest the existence of an ancient virus lineage leading to these last +ssRNA viruses and birnaviruses. However, similarities between the replication enzymes do not match similarities between the corresponding virus capsid maturation processes and genome arrangement, suggesting that re-assortment events occurred, that involved capsid and polymerase genes. The evolutionary links between +ssRNA viruses, birnaviruses and the other dsRNA viruses therefore constitute a complex issue that may help understanding the distinctive evolutionary pathways used by viruses to diversify and invade new hosts.

In this work, we sequenced the genome of a virus that infects the rotifer *Brachionus plicatilis* and that was previously identified as a Rotifer birnavirus (RBV) from biochemical and microscopy criteria [19]. Starting from the RBV sequence, we revisited the evolution of the *Birnaviridae* and their evolutionary affinities with +ssRNA viruses using advanced phylogenetic methods.

## Methods

### Cloning and sequencing the RBV genome

Viral RNA extraction, cDNA synthesis, DNA amplification and cloning in pGEM-T easy vector (Promega) were carried out as previously described [10]. Several overlapping clones covering segments A and B were sequenced.

### Protein sequences of birnaviruses

The genomes of a number of birnaviruses, infecting a wide range of hosts, have been sequenced. Birnaviruses possess bi-segmented double-stranded RNA (dsRNA) genomes. Segment B contains a single open reading frame encoding VP1, an RNA-dependent RNA polymerase (RdRp). Segment A encodes a polyprotein, pVP2-VP4-VP3. This polyprotein is processed to give the mature capsid protein VP2, a Ser/Lys protease and a multifunctional protein VP3 that interacts with the dsRNA to make filamentous ribonucleoproteins. RdRp is the only enzyme conserved in all known viruses (except satellite viruses) and thus it is often used to estimate virus phylogenies. However, in this work, to study the phylogeny of the birnaviruses, we built and analyzed trees for the four above proteins: VP1, VP2, VP3 and VP4.

### Available 3D structures of +ssRNA and dsRNA virus families

Table 1 shows a list of all the 3D structures of the RNA dependent RNA polymerase and capsid proteins for +ssRNA and dsRNA virus families available in the PDB. We added to this list, viruses for which no 3D structure is available such as *T*. *assigna* and *E*. *elaeasa* that belong to the *Permutotetraviridae* family and the drosophila A virus which is highly divergent from previously described viruses and which has not been assigned officially to a family yet.

**Table 1:** **Availableprotein 3D**-**structures of** +**ssRNA and dsRNA virus families**

**Virus**	**Family**	**Nucleicacid**	**Genome**	**RdRp**	**Capsidprotein PDB ID**	**RNP**	**Protease**
				**PDB ID**	**( *Triangulation *)**	**PDB ID**	**PDB ID**
Infectious bursal disease virus	*Birnaviridae*	dsRNA	bipartite	2pgg	1wcd (*T* = *13*)	2r18	
Infectious pancreatic necrosis virus	*Birnaviridae*	dsRNA	bipartite	2yi9	3ide (*T* = *13*)		2pnm
Blotched snakehead virus	*Birnaviridae*	dsRNA	bipartite		(*T* = *13*)		2gef
Tellina virus 1	*Birnaviridae*	dsRNA	bipartite				3p06
Bacteriophage?6	*Cystoviridae*	dsRNA	tripartite	1uvj	(*T* = *13*)		
Black beetle virus	*Nodaviridae*	+ssRNA	bipartite		2bbv (*T* = *3*)		
*N*. *capensis* Ω virus	*Alphatetraviridae*	+ssRNA	bipartite		1ohf (*T* = *4*)		
*T*. *assigna*, *E*. *elaease* viruses	*Permutotetraviridae*	+ssRNA	monopartite		(*T* = *4*)		
Drosophila A virus	*Unassigned*	+ssRNA	monopartite		(*T* = *3*)		
Poliovirus	*Picornaviridae*	+ssRNA	monopartite	2ijf	1hxs (*T* = *3*)		
Foot and mouth disease virus	*Picornaviridae*	+ssRNA	monopartite	2ec0	1bbt (*T* = *3*)		
Human rhinovirus type 14	*Picornaviridae*	+ssRNA	monopartite	1xr7	1aym (*T* = *3*)		
Norwalk virus (gastroenteritis)	*Caliciviridae*	+ssRNA	monopartite	1sh0	1ihm (*T* = *3*)		
Rabbit haemorrhagic disease virus	*Caliciviridae*	+ssRNA	monopartite	1khv	(*T* = *3*)		
Hepatitis C virus NS5B	*Flaviviridae*	+ssRNA	monopartite	2giq			
Bovine viral diarrhea virus	*Flaviviridae*	+ssRNA	monopartite	1 s48			

### Multiple sequence alignment strategy

To build the phylogenetic trees we need multiple sequence alignments of the corresponding proteins. Birnavirus protein sequences are sufficiently similar (about 25–35% sequence identity) to be aligned with usual multiple sequence alignment methods. We thus used two state-of-the-art programs: clustal-Ω [20] and MAFFT L-INS-I [21] which are based on different principles. We only retained amino-acid columns that were identical in both resulting multiple sequence alignments (consensus alignment).

By contrast, RdRp and capsid proteins of dsRNA and +ssRNA viruses exhibit very low sequence similarity and, capsid proteins, for instance, have complex domain organizations in which similar domains are intermixed in a complicated way with distinct domains. Therefore obtaining valid multiple sequence alignments can be quite challenging. To overcome this problem we used multiple structure alignments, when available, as a basis for multiple sequence alignments.

We first performed pair-wise structural alignments with VAST [22]. Using these pair-wise structural alignments we computed a multiple structural alignment (unpublished data) that delineated regions that could be superimposed in *all* the three-dimensional (3D) structures. This defines conserved regions (blocks), often termed the structural core, that are characteristic of the protein family fold. When the analyzed 3D structures correspond to remote homologs, mapping the 3D alignment to a sequence alignment is often tricky since a small inaccuracy in the 3D structure superimposition can lead to the assignment of a residue of a sequence with the neighbor of the true corresponding residue in the other sequence. This problem is exacerbated when one is considering the mapping of pair-wise structure alignments to a multiple sequence alignment and the resulting multiple alignment columns may contain residues that are not all equivalent (i.e., not all descending from a common ancestor). To circumvent this problem, we made the assumption that the most likely multiple sequence alignment *in the neighborhood* is the one that locally maximizes the cumulative score for the alignment. We thus performed a local sequence optimization to maximize the BLOSUM30 score of the corresponding sequence block by systematically shifting all the sequences by −2, -1, 0, +1, +2 residues.

To check the significance of these scores, we randomly generated blocks with an identical size using the same sequences. We performed on these randomly generated blocks the above optimization procedure and we recorded the scores. We then fitted a type 1 (Gumbel) extreme value distribution on these data using a maximum likelihood estimate function in MATLAB. This allowed us to verify that the scores we obtained for the original sequence blocks were indeed significant and not solely a consequence of our optimization procedure. This procedure defined a multiple sequence alignment made of a number of blocks with locally optimized scores (called the seed alignment).

Then two cases arose:

1. The 3D structure of a protein (the query) was not available but its sequence was sufficiently similar to the sequence of one of the proteins whose 3D structure had been solved (the target). We aligned the query sequence with the target sequence using a regular sequence alignment program. It was easy, then, to integrate, by transitivity, this query sequence into the seed alignment, since both alignments had the target sequence in common.

2. The most difficult case occurred when the 3D structure of the query protein was not known and its sequence did not share a clear similarity with any sequence of the seed alignment. In such a case, we generated for the query protein a multiple sequence alignment with PSI-BLAST [23]. This multiple sequence alignment was edited to avoid pairs of sequences with more than 70% identical residues. We then performed an alignment of the previously defined blocks (seed alignment) with the query multiple sequence alignment using a dynamic programming algorithm. Gap penalties were chosen such as preventing indels in the blocks. This resulted in an alignment of the blocks on the multiple sequence alignment generated with the query sequence, each block having a particular score. To estimate the reliability of the positioning of the blocks on the query multiple sequence alignment, we shuffled the columns of the latter and realigned the blocks with the resulting multiple sequence alignment. We carried out this shuffling procedure 200 times and collected the scores of the aligned block. As above, we fitted a Gumbel extreme value distribution on these scores for each block. This allowed us to estimate the significance of the score of each block by computing the probability that a particular block had a score equal to or larger than the observed value, just by chance. We tested the validity of this procedure with the nine RdRps for which the 3D structure is known using a jack-knife, i.e., we selected, in turn, height structures to build the blocks as described above and used the ninth one as a query. This test showed that we were able to correctly predict the position of the blocks along the query sequence for scores having a probability less than 0.01. On the other hand, we observed false negatives, that is, blocks correctly positioned along the query sequence but having a probability much larger than 0.01. Most of the time, this occurred when the block was located between two other blocks with large and significant scores that constrained the number of available positions for the former block.

All multiple sequence alignments have been deposited in TreeBASE.

### Phylogenetic analyses

Since our proteins are highly divergent, we paid special attention to choosing an appropriate evolution model: we examined site-heterogeneous and branch heterogeneous models in addition to standard ones. This also explains why we considered only likelihood-based (maximum likelihood and Bayesian inference) reconstruction methods. We first used ProtTest [24] to determine the most appropriate evolution model among a set of standard candidates models. We used the Bayesian Information Criterion (BIC) to select the model. For all datasets, ProtTest selected LG + I + G or variations of it (LG is an evolutionary models for proteins developed by [26]). We then compared LG + I + G to various mixtures model tailored to the evolution of divergent proteins. Those models are CAT20, which assumes that each position belong to one of 20 categories, each characterized by a specific stationary amino-acid distribution and structure-based models that assigns positions to one of 2 to 6 categories, according to the secondary structure of the protein, and use a different substitution matrix for each category. CAT20 and structure based models account for heterogeneity across sites in a much more specific way than standard rates across sites models.

Multiple sequence alignments constructed as above were analyzed using both maximum likelihood (PhyML [25] for standard model and phyml-cat [26] and phyml-structure [27] for structure based models) and Bayesian inference (MrBayes [28]) methods.

In ML analyses and to mitigate the effect of the starting tree, we used a full SPR search strategies with 5 random starting trees in addition to the default BIONJ starting trees. We included a proportion of invariable sites, optimized by the program (−v e), and a RAS modeled with a Gamma distribution with 4 categories with shape parameter optimized by the program (−c 4 -a e). For all alignments considered, at least 4 of the 5 random starting trees led to the same final tree as the BIONJ starting tree. Moreover, this shared tree was always the overall ML tree.

For MrBayes, we used the LG + G + I model, as selected by ProtTest. We used a gamma-shaped distribution of rates across sites, uniformly distributed on the interval [0,200]; the proportion of invariable sites was uniformly distributed on the interval [0,1]; the Gamma distribution was approximated using 4 categories; all topologies were equally probable a priori; the branch lengths were unconstrained.

Structure-based models such as CAT20, EX, EHO and EX_EHO are not implemented in MrBayes and therefore we did not infer topologies using these models under Bayesian inference. However, MrBayes allows within site heterogeneity with covarion models. We therefore ran chains under a LG + G + I and covarion model and compared the two models (with or without covarion) using Bayes factor. The covarion model was not only slower to converge but also rejected by Bayes factor (bayes factor > 100 in favor of LG + G + I). Therefore, we only provide results obtained without covarion.

All trees and multiple sequence alignments have been deposited in TreeBASE. (http://purl.org/phylo/treebase/phylows/study/TB2:S13955).

### Analysis of recombination events in segments A and B of birnaviruses

We implemented the PDM method proposed by [29] (since TOPALi which incorporates this method does not allow the use of amino acid sequences for recombination breakpoint location analyses). As other recombination analysis methods, PDM is based on the principle that phylogenetic relationships derived from different regions of a multiple sequence alignment will be similar when no recombination has occurred. In practice, a window of length L is moved along the multiple sequence alignment by increment of ΔN positions (we used L = 100 aa and ΔN = 5 aa). At each position, the posterior probability distribution of the tree topologies, conditional to the multiple sequence alignment in the window, is computed. For this purpose, we employed MrBayes, with 120,000 steps of MCMC and a LG + G + I model. In the absence of recombination, one expects the posterior probability distributions computed from different windows to be similar. This similarity is quantified by the Kullback–Leibler (KL) divergence between the distributions. In [29], the authors, instead of using the individual tree topologies, first clustered these topologies using a Robinson-Fould distance, and then computed the posterior probability distribution of the clusters conditional to the multiple sequence alignment in the window. This reduced the dispersion of the posterior distributions, providing more robust results. We exactly followed the procedure described in their paper. We, thus, also implemented the procedure Husmeier and coll. proposed for estimating the significance of the observed peaks in the KL divergence plot. However, we found that this procedure clearly underestimated the significance thresholds.

Therefore, to estimate empirically the significance of the peaks found in the KL divergence plot we carried out simulations using Seq-Gen [30]. Seq-Gen is a program for simulating the evolution of nucleotide or protein sequences along a phylogeny, according to a particular model of substitution. We concatenated the multiple sequence alignments for the VP1, VP2, SPC, VP4 and VP3 birnavirus proteins of the eight taxa (SPC corresponds to the region between VP2 and VP4 that is part of the pro-VP2 polypeptide chain). Starting with each taxon concatenated amino acid sequence, in turn, we generated 13 multiple sequence alignments sets using the tree obtained for one of the above windows with MrBayes. This resulted in 104 sets of eight 897-amino acid long sequences. Since the LG substitution matrix is not available in Seq-gen we used the Blosum62 matrix. For other parameters, such as the proportion of invariable sites, the shape of the Gamma rate heterogeneity, etc. we used the values estimated by MrBayes for the selected window. For each of these 104 sets, we carried out the KL divergence computations described above and we recorded the height of the maximum peak that was found. This provided us with a non-parametric estimate of the significance thresholds (99, 95 and 90 confidence levels).

## Results

### Sequence of a rotifer birnavirus (RBV) genome

The rotifer *Brachionus plicatilis* is commonly cultivated for feeding the fry of marine fish in hatcheries in production tanks. After a population collapse, a virus was detected by electron microscopy and isolated from dying plankton [19,31,32]. This virus showed the typical features of a birnavirus, with non-enveloped, single-shelled icosahedral virus particles of approximately 60 nm in diameter and displaying a bi-segmented double stranded RNA genome with a size of about 2 × 3 kilobases. We sequenced the two genomic segments of the birnavirus previously isolated from *Brachionus plicatilis*. The ORF present in the larger genomic segment (segment A) consists of 3180 nucleotides (nt) encoding a polyprotein of 1060 amino acids (aa) sharing 20-30% identity with its birnavirus pVP2-VP4-VP3 homologs. No additional ORF was evidenced on this segment. The ORF in segment B consists of 3135 nt encoding a 1045 aa protein displaying 19-29% sequence identity with its birnavirus replicase homologs (Figures 2A and B). RBV thus appears as a new member of the Birnaviridae family that is divergent from other birnaviruses infecting vertebrates, mollusks or arthropods (GenBank accession numbers for segment A: FM995220, and segment B: FM995221).

### Primary structures of the polyprotein and RNA polymerase RBV

On the basis of experimentally characterized cleavage sites in other birnavirus polyproteins, the RBV polyprotein maturation cleavage sites were identified by sequence homologies (Additional file 1: Figure S1). The primary cleavage sites at the pVP2-VP4 and VP4-VP3 junctions may occur between residues 529–530 and 773–774, respectively and three additional cleavage sites were predicted in the pVP2 C-terminal domain (at amino acids positions 467–468, 511–512 and 518–519). The predicted peptides have homologs in other birnaviruses. Overall, the structure of the VP2 S domain appears well conserved, but the length of the β-strands and the loops constituting the P domain appear to be very different when compared to their IBDV and IPNV homologues. The VP3 sequences have deeply diverged during birnavirus evolution, suggesting that the structural constraints are less restrictive than for the capsid protein. Similarly, only a few residues are conserved between the RBV VP4 and other birnavirus VP4, including the catalytic site defined by the serine hydrolase GxS signature (Ser at position 673). Overall, based on sequence alignments, the 3D-structures of VP2, VP3 and VP4 of RBV are expected to be rather similar to their birnavirus homologs.

Concerning the RBV VP1, a striking feature of the sequence is the presence of a large C-terminal domain only found in its DXV and ESV homologues otherwise, which may suggest a closer proximity of these three viruses. Furthermore, the RBV VP1 sequence revealed the conservation of the unique C-A-B motif arrangement in the palm subdomain of the RBV replicase, a hallmark that is shared by birnaviruses and the permutotetraviruses [33,34]. This motif rearrangement is a result of a topological relocation of the protein main chain between two internal positions, separated by about 110 aa.

Thus, the sequences of the RBV proteins identify this virus as a new member of the *Birnaviridae* family with a typical genomic structure, defining a new genetic cluster in this family. Using a phylogenetic approach, we undertook a study of the evolutionary links among birnaviruses, including RBV and the birnaviruses recently described from insects.

### Analysis of potential re-assortment/recombination genomic events

**Figure 3:**
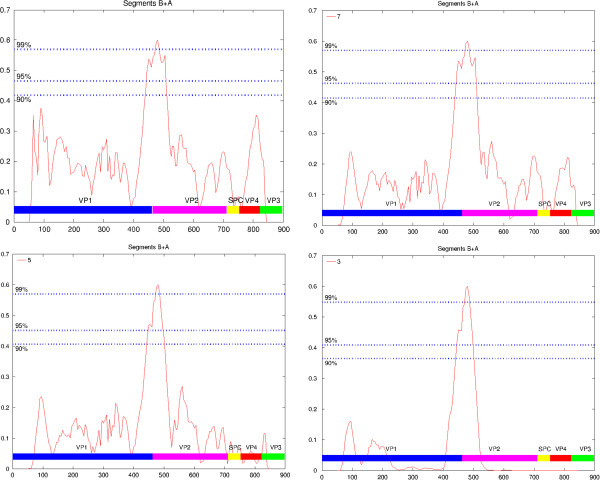
**Plots of the Kullback–Leibler divergence along a concatenation of the segment proteins.** The abscissa axis displays the residue numbers in the concatenated alignment. The corresponding proteins, in the order used, are shown at the bottom of the plot in different colors. The ordinate axis shows the KL divergence value which varies between 0 and 1. Peaks are indicative of possible recombination breakpoint locations. Dotted lines show the significance thresholds (90, 95 and 99 confidence levels). The number of clusters used for the analysis is indicated in the top left corner of each plot.

The supermatrix is a common approach to analyse data from multiple genes (proteins). It consists in concatenating the individual genes (proteins) to form a single supergene (superprotein) to which usual phylogenetic inference methods are applied. However, this approach assumes that all or at least most of the genes have roughly the same phylogenetic history. This might not always be the case for highly plastic genomes, such as those of dsRNA viruses, which are potentially subject to segment re-assortments or recombination events. To test the possibility of genomic rearrangements we concatenated the multiple sequence alignments for proteins of both the A and B segments (pVP2, VP3, VP4 and VP1) in different orders and analysed the resulting multiple sequence alignments for potential recombination breakpoints using PDM [29] as described in Methods. Figure 3 presents the plot of the Kullback–Leibler divergence for different values of the number of tree topology clusters. Peaks in this plot are indicative of the location of possible recombination events. The dotted lines indicate the significance of the peaks. For instance, we determined empirically that 99% of the peaks have a height that is less than 0.57 if there is no recombination (see Methods for details of the procedure). Consideration of Figure 3 (and other plots with different orders of the proteins, data not shown) reveals that the only significant peak is the one located between VP1 and proteins of the segment A, here VP2. In other words, it is highly probable that the two segments have been re-assorted in the different lineages resulting in different phylogenetic histories. This was confirmed by an AU test [27] of topologies. We inferred topologies TA and TB independently on segments A and B. The AU test rejects topology TB for segment A (p = 0.03), but not topology TA for segment B (p = 0.12). However, it should be noted that the AU test is quite conservative. With regard to these results, in the subsequent phylogenetic analyses, we will consider VP1 on the one hand and a concatenation of VP2, VP3 and VP4 on the other hand.

### Selection of an evolutionary model for virus protein evolution

Since the phylogenetic analysis of virus proteins may be very difficult due to their fast and extensive divergence, using an appropriate model of amino acid replacement is critical to study RNA virus protein evolution and phylogeny. We used ProtTest [24] to select the best evolution model for our proteins. Among the models available in ProtTest, LG + G + I or variations of it (LG + I + G + F, LG + G) were selected for all datasets. It consists of the LG amino-acid substitution matrix supplemented with a fraction of invariable sites (+I) and rate heterogeneity across sites (RAS) model, modelled with a discrete Gamma distribution with four categories. In addition to this model, we explored various models specifically designed for divergent proteins. The first, CAT20 [26] assigns each position of the alignment to one of 20 pre-computed categories. Each category is characterized by a specific profile of amino-acid stationary frequencies. Within a category, changes are biased towards amino acids with high stationary frequency, so that the amino acids observed at a given position are but a subset of all amino acids. Amino acids with high probability within a category often share one or more properties: electric charge, hydrophobicity, aromatic character, etc. However, apart from their specific profiles, all sites share a common substitution matrix: the F81 (also called Poisson) matrix. The second model, STRUCTURE [35], is a mixture model where each position is assigned *a priori* to a category, based on the secondary structure of the protein at that position. All sites of a category evolve according to the same substitution matrix. However and unlike CAT, the categories differ not only on their stationary distribution but also on their (symmetric) substitution matrices. Three classification schemes were tested: Exposed against Buried (EX, 2 categories), Extended, Helix and Other (EHO, 3 categories) and a combination of the two previous schemes (EX_EHO, 6 categories). The CAT and STRUCTURE models are fundamentally different from standard models included in ProtTest because they are mixture models: each category has a distinct substitution matrix. In standard models, the only heterogeneity across sites comes from the RAS: slow-evolving and fast-evolving positions share the same substitution matrix, up to a scaling factor coming from the Gamma distribution. In CAT and STRUCTURE models, no such linkage exists between substitution matrices of different categories. Furthermore, CAT and STRUCTURE also include a RAS component (+G) and a proportion of invariable sites (+I) to account for different rates of evolution. We compared LG + I + G to CAT + I + G and (EX/EHO/EX_EHO) + I + G using AIC and BIC. Both criteria selected a structure-based (EX_EHO) model with a gain of BIC per position ranging from 0.48 (segment A) to 0.53 (segment B). We therefore performed all subsequent analyses with the EX_EHO model. We should however mention that all models inferred the same topology with only slight differences in branches lengths and thus that the results presented below are robust to the choice of an evolution model.

### Phylogenetic analysis of birnaviruses

Given the phylogenetic position of rotifers among the birnavirus hosts, we were interested to get more insight into the affinities of RBV with the other birnaviruses described so far. We thus analyzed the phylogenetic relationships between proteins of segment A (concatenation of VP2, VP3 and VP4 proteins) and segment B (VP1 protein) for the eight birnaviruses described so far using the selected evolutionary model described above.

#### Phylogeny of segment B (VP1 protein)

**Figure 4:**
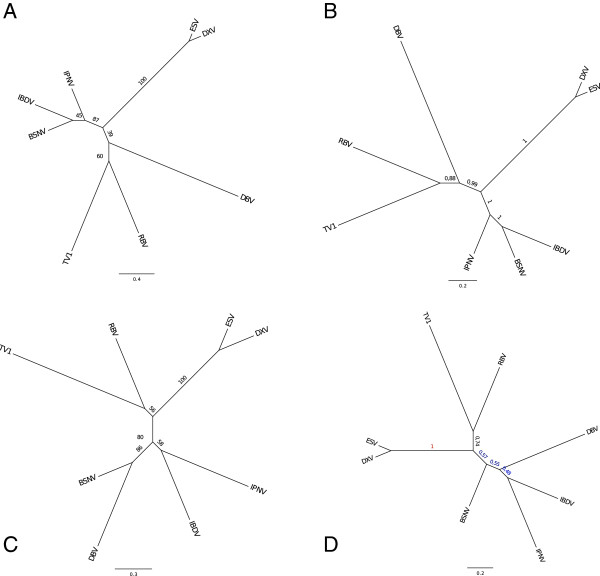
**Phylogenetic trees for VP1 and VP2 + VP3 + VP4 proteins of eight birnaviruses.** Inset **A**: maximum likelihood (ML) tree for VP1 proteins (PHYML); inset **B**: Bayesian consensus tree for VP1 proteins (MrBayes); inset **C**: maximum likelihood tree for the concatenation of VP2, VP3 and VP4 proteins (PHYML); inset **D**: Bayesian consensus tree for concatenation of VP2, VP3 and VP4 proteins (MrBayes). Figures along the branches are the bootstrap values and the posterior probabilities for respectively the ML trees and Bayesian consensus trees. Distance scales are displayed below the plots.

Figures 4A and B presents, respectively, the maximum likelihood tree using PHYML and the consensus tree using MrBayes for VP1. Both trees are congruent. The partition between vertebrate viruses and other viruses is well supported with, respectively, a bootstrap value (bv) of 87/100 and a posterior probability (pp) of 1. RBV groups with TV-1 unambiguously (bv = 60, pp = 0.88). This group clusters first with the drosophila virus, DBV, (bv = 39, pp = 0.99) then with the group formed by the second drosophila (DXV) and the mosquito (ESV) viruses. Notice that arthropod birnaviruses do not form a clade. VP1 sequences of DXV and ESV are very similar (69% sequence identity). By contrast, the two Drosophila virus VP1 sequences are very different. They share 22.5% sequence identity that corresponds to the smallest pairwise similarity of all VP1 proteins. This part of the virus tree is congruent with the corresponding host tree. Regarding the VP1 of vertebrate viruses, the avibirnavirus IBDV always clusters with BSNV, a fish birnavirus (genus *blosnavirus*). Table 2 shows the percentages of sequence identity between these three taxa for the four proteins. It is striking that the maximum value is always found for IBDV and one of the fish viruses, whereas the minimum value is usually found for the two fish viruses (except for VP1).

**Table 2:** Percentage sequence identity for vertebrate birnavirus

	**IBDV**	**IPNV**	**BSNV**		**IBDV**	**IPNV**	**BSNV**
IBDV		*45*.*3*	**49**.**3**	IBDV		**36**.**0**	35.2
IPNV	42.9		45.9	IPNV	22.3		*31*.*0*
BSNV	**48**.**1**	*41*.*7*		BSNV	**24**.**5**	*21*.*3*	

VP1 (left, upper triangular matrix) VP2 (left, lower triangular matrix), VP3 (right, upper triangular matrix) and VP4 (right, lower triangular matrix). The maximum value is shown in bold, the minimum value in italic.

A common characteristic of all the trees we built with MrBayes and PHYML is that i) MrBayes is always more certain about the topology than is PHYML and ii) distances estimated by PHYML are always larger than those calculated by MrBayes (for the same tree topologies). It is known that Bayesian probabilities are on average higher than nonparametric bootstrap support values although direct comparison of the two measures is difficult [36]. Bootstrap is generally thought consistently conservative, as bootstrap proportions tend to underestimate the probability of the clades to be true, especially when this probability is high [36]. Bayesian inference, as any parametric method, is sensitive to model assumptions. Therefore, in the following, we consider that the two measures provide lower and upper bounds of the true value.

#### Phylogeny of segment A (VP2 + VP4 + VP3 proteins)

Figures 4C and D displays, respectively, the ML tree and the Bayesian consensus tree for the concatenated VP2, VP3 and VP4 proteins. The two trees differ by the position of DBV that branches, in the ML tree with BSNV with a strong bootstrap support (bv = 86) and in the Bayesian consensus tree on the branch joining {IBDV, IPNV} and BSNV with a relatively weak support (pp = 0.48 and 0.55). As a consequence, the partition between vertebrate and invertebrate viruses is moderately supported in the Bayesian consensus tree (pp = 0.57). Indeed, when examining the topology of the five trees with the largest probabilities values (whose cumulative probability is greater than 0.90), one observes that DBV tends to cluster with: IPNV (pp = 0.445), TV1 (pp = 0.235), the cluster {TV1, RBV} (pp = 0.164), IBDV (pp = 0.053, the cluster {IBDV, IPNV} (pp = 0.050), the tree topology for the other taxa remaining the same (data not shown). Additional file 2: Figure S2 presents the tree that is obtained after removing the DBV sequence in the VP234 multiple sequence alignment. One obtains a single seven-taxon tree with a very large posterior probability (pp = 0.983). The subtree topology for the invertebrate viruses is congruent with that of their hosts but the subtree topology for vertebrate viruses is incongruent with that of their hosts, the avibirnavirus IBDV clustering with the fish birnavirus IPNV. All branches of this seven-taxon tree are strongly supported with all posterior probabilities greater than 0.99. Therefore, segment A of DBV appears difficult to classify precisely. For instance, in the above Bayesian trees, it never clusters with other insect viruses.

### Evolutionary affinities of dsRNA and +ssRNA viruses sharing common structural features

As structures of RdRp or capsid proteins have been solved for a number of birnaviruses and +ssRNA viruses, we then investigated evolutionary affinities of birnaviruses with +ssRNA viruses (the first evidence of a relationship between birnaviruses and +ssRNA viruses date back to 1988 [37]) using advanced phylogenetic methods and alignments based on structures.

#### Analysis of RdRp proteins

**Figure 5:**
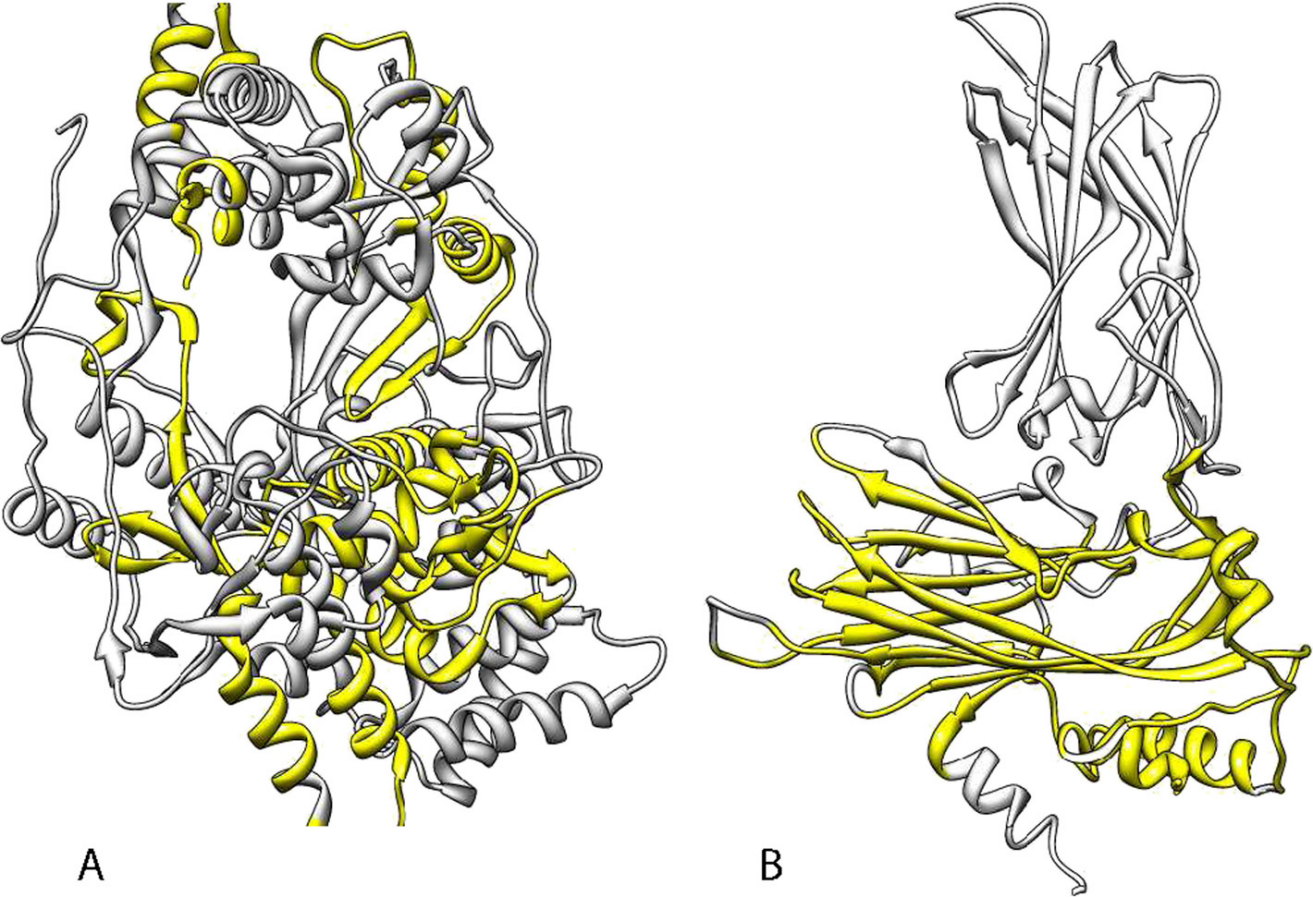
**Conserved regions in VP1 and VP2 structures.** Conserved regions identified by comparison of protein 3D structures are represented in yellow for the IBDV VP1 **(A)** and VP2 **(B)**. Sequences corresponding to these structurally conserved regions were extracted and used for multiple alignments and phylogenetic analysis.

The quasi-canonical RdRps of birnaviruses and the TaV/EeV viruses (*Permutotetraviridae*) constitute a separate, deeply rooted RdRp lineage that apparently evolved in parallel to all other viral and non-viral RdRps [33]. To clarify the origin of this RdRp lineage in the RNA virus world, we used all available relevant RdRp 3D-structures to produce structure-based alignments of birnavirus sequences with sequences of the most related +ssRNA viruses (noda-, alphatetra-, permutotetra- and Drosophila A viruses) and carried out phylogenic analyses. Sequences of RdRp proteins are poorly similar. However, the 3D structures of a number of RdRp of +ssRNA or dsRNA viruses (see Table 1) allowed us to define structurally conserved regions. Interestingly, one of the RdRp 3D structures solved is that of the bacteriophage PHI6 whose host is a prokaryote and thus constitutes a clear outlier with respects to other RdRp proteins. One can thus hope that the conserved regions so defined will be truly characteristic of the RdRp protein family from +ssRNA and dsRNA viruses. There are 14 structurally conserved regions (blocks) corresponding to 159 residues. Most of the identified sequence motifs in RdRps are associated with some of these blocks, e.g., motif G to block 3, motif F to block 5, motif A to block 7, motif B to blocks 9 and 10, motif D to block 11, motif E to block 12 and motif C to block 14 (the order of the motifs is irrelevant for phylogenetic analyses). Figure 5A shows the mapping of these conserved regions on the 3D structure of the IBDV RdRp. For viruses whose RdRp 3D structure was not available (see Table 1), we used the alignment scenarios described in the Method section. As can be seen on the multiple sequence alignment (available in TreeBASE), we could not align the 14 blocks with enough confidence (p < 0.01) on the corresponding query sequences, the worst case being the N. capensis ω virus sequence for which only 3 blocks could be confidently aligned. Hereafter, we will analyze trees with 9 species (those for which the RdRp 3D structure is available), 12 species (the previous ones plus the alphatetra-, permutotetra- and DAV viruses) and 13 species (adding the nodavirusRdRp). In the first two trees we will consider the topology of four groups of species, corresponding to the *Caliciviridae* family, the *Picornaviridae* family, the *Flaviviridae* family and a group labeled “others” that gathers dsRNA viruses in the case of the 9-species tree, and dsRNA viruses, alphatetra-, permutotetra- and DAV viruses for the 12-species tree). For the 13-species tree, the black beetle virus (*Nodaviridae* family) forms a group of its own.

**Figure 6:**
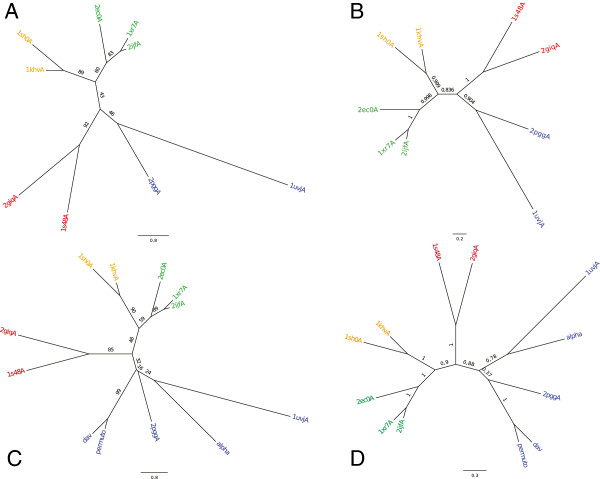
**Phylogenetic trees of RdRp proteins for +ssRNA and dsRNA viruses.** Inset **A**: maximum likelihood (ML) tree for the nine RdRps whose 3D structure is known for the virus families considered in the study. Leaves are labeled with RdRp PDB codes (1urjA: Bacteriophage phi6; 2ppgA: Infectious bursal disease virus; 1 s48A: Bovine viral diarrhea virus; 2giqA: Hepatitis C virus NS5B; 1sh0A: Norwalk virus; 1khvA: Rabbit haemorrhagic disease virus; 2ec0A: Foot and mouth disease virus; 1xr7A: Human rhinovirus type 14; 2ijfA: Poliovirus. See Table 1 for more information). Viruses of the *Picornaviridae* family are displayed in green, those of the *Caliciviridae* family in orange, those of the *Flaviviridae* family in red and others (here dsRNA viruses of the *Birnaviridae* and *Cystoviridae* families) in blue; Inset **B**: Bayesian consensus tree for the same nine viruses; Inset **C**; ML tree for the previous nine viruses plus three viruses whose RdRp 3D structure is not available: Drosophila A virus (dav), *N*. *capensis* Ω virus (*Alphatetraviridae*), *T*. *assigna* virus (*Permutotetraviridae*); Inset **D**: Bayesian consensus tree for the same twelve viruses. Figures along the branches have the same meaning as in Figure 4.

Figures 6A and B presents, respectively, the maximum likelihood tree (PHYML) and the Bayesian consensus tree (MrBayes) for the 9 species. Both trees are congruent and show a fair support (bv = 0.48 and pp = 0.84) for the partition {Calici, Picorna | Flavi, Others}. The three alternative topologies T1 = {Calici, Flavi | Picorna, Others}, T2 = {Calici, Picorna | Flavi, Others} which is also the ML tree and T3 = {Calici, Others | Flavi, Picorna} have likelihood −3494.03, -3491.85 and −3495.11. An AU test with these topologies almost rejects topology T3 (p = 0.065) but cannot discard topology T1 (p = 0.32). The bootstrap probabilities of these trees are 0.29, 0.69 and 0.02 indicating a clear but not significant preference for topology T2 (see Additional file 3: Figure S3). Additional file 4: Figure S4 shows an evaluation, with the program TREE-PUZZLE [38], of the internal branch support of the three possible topologies that can be generated with these four groups using likelihood mapping for visualizing the phylogenetic content of the multiple sequence alignment. The results of TREE-PUZZLE are consistent with the AU test.

As expected, 1ujvA, being a virus of prokaryote, exhibits the longest branch in all trees.

Figures 6C and D presents, respectively, the maximum likelihood tree (PHYML) and the Bayesian consensus tree (MrBayes) for the 12 species. The trees are congruent and show a slightly improved support for the same partition as above. The differences in likelihood between the three alternative topologies and results of the AU are also improved: T1, T2 and T3 have likelihood respectively −4121.14, -4118.16 and −4122.31. The AU test rejects topology T3 (p = 0.05) but not topology T1 (p = 0.28). Interestingly, the bootstrap probabilities of the three topologies are 0.25, 0.73 and 0.02 indicating a clear preference for T2 (the ML tree) although the difference between T1 and T2 does not reach significance (see Additional file 5: Figure S5). The three new sequences group together with the dsRNA viruses (in the “others” group). Interestingly, the drosophila A virus (DAV) that has been labeled as a picorna-like virus [39], clusters tightly with the RdRp sequence of the *T*. *assigna* virus that is a +ssRNA virus belonging to a newly defined *Permutotetraviridae* family [40]. In Figure 6D, this group further clusters with the birnavirus RdRp, 2pggA. Permutotetra-, DAV and 2pggA have permuted RdRps whereas all other viruses have canonical RdRps. The tree shown on Figure 6D confirm the claim of [33] that permuted RdRps form a separate lineage in the polymerase tree. However, the ML tree of Figure 6C does not support this view since 2ppgA clusters with the viruses of the *Alphatetraviridae* and *Cystoviridae* families, although with quite an insignificant bootstrap value of 16. Indeed, in Additional file 6: Figure S6, the ML tree for 13 species groups together taxa with permuted RdRps as in Figure 6D with a bootstrap value of 42. Interestingly, the two dsRNA viruses (from the *Birnaviridae* and *Cystovirida*e families) do not form a clade of their own but are clustered with other +ssRNA viruses.

Additional file 6: Figure S6 shows Bayesian consensus tree for 13 species. The most probable tree found in the MCMC simulation has a mere 0.08 probability and 136 trees are necessary to obtain a cumulative probability of 0.90. An analysis of these trees shows that the nodavirus taxon can branch almost anywhere on the 12-species tree, the resulting 13-species trees having similar small posterior probabilities. In the Bayesian consensus tree, the preferred position for the noda taxon is on the branch joining the clusters {Others, Flavi} and {Calici, Picorna}. The ML tree obtained with PHYML is congruent with the Bayesian consensus tree and shows weak support for the ML topology (data not shown). The AU test, as for the two datasets above, rejects topology T3 and keeps T1 and T2.

#### Analysis of capsid proteins

**Figure 7:**
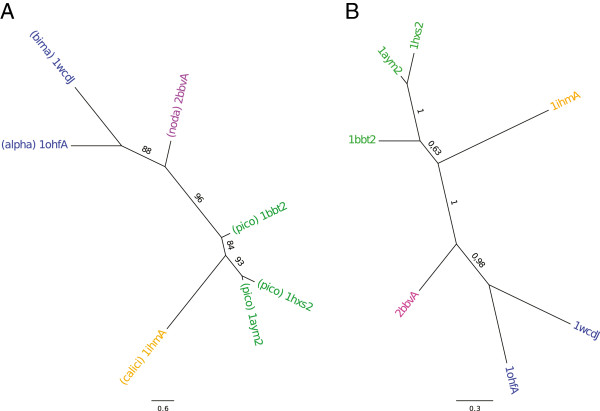
**Phylogenetic trees of capsid proteins for +ssRNA and dsRNA viruses.** Inset **A**: ML tree for the seven capsid proteins whose 3D structure is available (1wcdJ: Infectious bursal disease virus; 1ohfA: *N*. *capensis* Ω virus; 2bbvA: Black beetle virus; 1ihmA: Norwalk virus; 1bbt2: Foot and mouth disease virus; 1aym2: Human rhinovirus type 14; 1hxs2: Poliovirus. See Table 1 for more information). The same colors as in Figure 5 are used. Black beetle virus (*Nodaviridae* family) is displayed in magenta. Inset **B**: Bayesian consensus tree for the same seven proteins. Figures along the branches have the same meaning as in Figure 4.

We conducted a similar phylogenetic analysis on the capsid proteins of the same viruses, when possible. Table 1 shows the list of available capsid protein 3D structures of dsRNA and +ssRNA viruses in the PDB. Preliminary results (at 7.5 Å resolution) for the nucleocapsid of the bacteriophage phi6 suggest that the birnavirus capsid protein has no equivalent in the bacteriophage PHI6 genome [41]. Low resolution 3D structures of the capsid of viruses from the *Flaviviridae* family [42] tend to indicate that proteins involved in building the capsid have no evolutionary relationship with birnavirus VP2 proteins. As for RdRp proteins, we carried out pair comparisons of the seven available structures of capsid proteins (Additional file 7: Figure S7), generated a multiple structure alignment from which we obtained a multiple sequence alignment as described in the Method section. This resulted in a multiple sequence alignment having eight blocks (mapped on the 3D structure of IBDV capsid, see Figure 5B) and a total length of only 85 residues (seeTreeBASE). We then tried to align these blocks on the sequences of the capsid proteins for which no 3D structure was available (Drosophila A virus, *T*. *assigna* virus). For DAV and for *T*. *assigna* virus, respectively, no block and one block could be significantly aligned with a probability < 0.01. Therefore, we only built the phylogenetic trees for the seven species whose capsid 3D structure was available. Figures 7A and B presents the ML tree obtained with PHYML and the consensus tree obtained with MrBayes. The Bayesian consensus tree shows a clear partition {Others, Noda | Calici, Picorna}. The topology is well supported with large posterior probability values. The ML tree is almost similar, but the virus of the *Caliciviridae* family branches among the viruses of the *Picornaviridae* family (the bootstrap values are significant).

## Discussion

In this work, we determined the full-length genomic sequence of a virus previously identified from the rotifer *Branchionus plicatilis* as a birnavirus on the basis of morphological features. Both genomic organization and protein sequences encoded by ORF definitely confirm that RBV is a typical birnavirus, thus adding a seventh genetic cluster to the *Birnaviridae* family. Rotifers are small zooplanktonic animals from fresh- and seawater, characterized by a ciliated anterior corona used for locomotion and food gathering, and a pharynx with a complex system of jaws. Their large population size and high turnover rate make rotifers an important component of food webs since they are eaten by invertebrate predators and fish fry. Interestingly, the taxonomic affinities of these species remain controversial despite long standing efforts. A recent global phylogenetic analysis of metazoa based on large sample of EST and genomic sequences showed that rotifers – together with Gastrotrichs and Gnatostomulids – group with Platyhelminthes more than with any other stable groups of animals and placed them among the Lophotrochozoa [43]. The presence of a birnavirus in a rotifer therefore extends significantly the host range of this viral family to a fourth phylum in addition to mollusks, arthropods and chordates. Thus, we conducted a study to confront the tree of birnaviruses with the tree of their hosts.

Our analysis showed clearly that the birnavirus segments A and B followed different evolution pathways, due to likely re-assortments. It is worth mentioning that within IPNV strains, segments A and B re-assortment has been evidenced in wild fish [44] and that for IBDV, expansion of the very virulent strains has been postulated being associated to segment B re-assortment in the late 1980s [45]. Independent analysis of proteins encoded by segments A and B therefore produced trees of viral sequences, which we compared to the tree of the host species. While virus protein trees are generally well congruent with the host species *at the phylum level*, discrepancies appear in the detailed structure of the branch *within phyla*, possibly reflecting specific adaptations of host-pathogen interactions. Thus, neither segment B nor segment A analyses led to a tree compatible with the structure of the vertebrate phylum, since the avibirnavirus IBDV always clustered with one of the fish birnaviruses. Similarly, within arthropod birnaviruses, the DBV sequence does not classify at all according to host taxonomy: for segment B, it constitutes a single branch that does not group with the sequence of the two other insect birnaviruses, while for segment A it even falls in the vertebrate birnavirus cluster. These observations would indicate that birnaviruses host specificity may be less narrow than what is observed with avian birnaviruses (IBDV) and may contribute to re-assortment between phylogenetically distant birnaviruses. Genome characterization of new birnaviruses, specially infecting insects and invertebrates may help understanding re-assortment plasticity in birnavirus genomes.

To further investigate the origin of the birnavirus lineage and of its bipartite RNA double-stranded genome, we carried out a comprehensive phylogenetic analysis of protein regions selected from 3D-structure conservation, for both capsid and RdRp of RNA viruses. In the early nineties, Koonin and Dolja proposed hypothetical evolutionary scenarios for +ssRNA viruses based primarily on a tentative phylogeny of the RdRps and also on the gene order conservation in the different genomes [46]. The phylogeny analysis was based on multiple sequence alignments of relatively short functional motifs and the generation of the trees was carried out with distance (UPGMA and least square methods, FITCH and KITSCH) and parsimony methods implemented in the Phylip package [47]. A couple of years later, Zanotto et al. [48] showed that the sequence similarities and phylogenetic signal were insufficient to support many of the proposed evolutionary groupings of RNA viruses. Almost two decades later, the 3D structures of a number of RdRps and capsids from different dsRNA and +ssRNA virus families are now available (see Table 1). Computational molecular evolution techniques have also significantly progressed with the introduction of new approaches based on nucleic acids and amino acids’ substitution models that integrate powerful statistical techniques such as maximum likelihood or Bayesian methods to evaluate the likelihood of the data given the model parameters or the posterior probability of the generated trees. It thus seemed timely to us to try re-assessing the evolutionary relationships between the dsRNA and +ssRNA virus families whose RdRp 3D structure is available. Our goal was to use these 3D structures to obtain reliable multiple sequence alignments comprising more residues than the ones based only on sequence alignments.

The combination of robust sequence alignments based on 3D structure alignments and the use of advanced phylogeny techniques, in particular site-heterogeneous evolutionary models, allowed us to overcome the difficulties due to the very divergent sequences in the different virus families [49] and to obtain a more precise picture of the relationship between families of +ssRNA and dsRNA viruses. We also carried out tests with models of heterotachy but they provided less convincing results in terms of likelihood of the trees (data not shown). Although the SH test cannot distinguish topologies T1 and T2 at a 5% significance level, several lines of evidence indicate that the T2 topology that groups {others, flavi-} and {picorna-, calicivirus} is the more likely. We must emphasize that the SH test is well known to be extremely conservative.

Trees in Figure 6 and Additional file 6: Figure S6 tend to confirm the claim of [33] that viruses with permuted RdRps belong to a common evolution lineage. We believe that the topology shown in Figure 6C which clusters the birnavirus taxon with viruses of the *Alphatetraviridae* and *Cystoviridae* families (see Results section) is an “accident”. ML methods single out the best tree. It might exist other trees with slightly different topologies that are equally good in terms of likelihood, i.e., whose likelihood value is not significantly smaller than the best one. Notice also that phylogenetic methods do not take into account the fact that RdRps of the birna-, permutotetra- and Drosophila A viruses share a common rare evolutionary event, namely the permutation of the functional ABC sequence motifs that involve a local rearrangement of the polypeptide chain. However, this relative uncertainty in precisely classifying the birnavirus taxon certainly reflects a true phenomenon. Birnaviruses, according to their RdRps, are evolutionary closer to DAV and permutotetraviruses than to alphatetra- and cystoviruses. In contrast, judging from their capsid protein, birnaviruses appears closer to the alphatetra- and nodaviruses (the bacteriophage PHI6 virus of the *Cystoviridae* family has a different capsid organization). It would have been interesting to confirm (or invalidate) this point by including the capsid sequences of the DAV and permutotetraviruses in the analysis but, as explained above, we were not able to obtain convincing alignments for these virus proteins. Also, birna-, noda- and alphatetraviruses share the same bipartite genome organization whereas DAV and the permutotetraviruses have a monopartite genome organization (and the bacteriophage PHI6 virus has a tripartite genome organization). Therefore, birnaviruses appear to be intermediary between noda- and alphatetraviruses on the one hand and permutotetraviruses and DAV on the other hand although they are unique in having an RNA double-stranded genome. Inspection of the trees in Figures 6C and D reveals that the dsRNA viruses do not form a clade, they are intermixed with +ssRNA viruses. The transition between dsRNA and +ssRNA (or conversely) does not give the impression of being a rare evolutionary event (dsRNA viruses have been shown to cluster with viruses of the alpha-like superfamily [50] and of the picorna-like superfamily [51]). This might explain, in part, the surprising observation that there exist two birnaviruses in Drosophila (DXV and DBV) that do not seem closely related, neither considering segment A nor segment B. One could hypothesize that new birnaviruses are created by associating an RdRp, as segment B, from a virus of the *Permutotetraviridae* family or a similar family (DAV has not yet been established as belonging to the *Permutotetraviridae* or to a new family having similar characteristics) and a segment A belonging to a *Nodaviridae*, an *Alphatetraviridae* or another similar family. Our finding that segments A and B have different evolutionary histories offers support to this hypothesis. Of course, this scenario requires an explanation of how the monopartite genome of the *Permutotetraviridae* family would be split so as to only keep the part corresponding to the RdRp molecule as segment B. Gibbs and colleagues have suggested that dsRNA replicons from plant might have evolved from a single-stranded RNA virus lacking the coat protein [50]. It has been shown that the lack of a coat protein affects the balance between viral positive-sense and complementary-sense RNA strands (the coat protein up-regulating the plus strand synthesis and down-regulating the minus strand synthesis [52]). However, the precise mechanism by which the newly created birnavirus eventually would acquire a double-stranded genome remains elusive.

## Conclusions

In this work, we have analyzed the evolutionary relationship among birnaviruses of a wide range of hosts. We showed that segments A and B are very likely to have been re-assorted in this family and thus have a different evolutionary history. Using these two segments, we also showed that there is a good correlation between the phylogeny of the viruses and that of the hosts at the phylum level. However, the situation is much less clear when one studies evolutionary relationships within the phyla. This is the case for vertebrate viruses in which the avibirnavirus alternatively clusters with one of the two fish birnaviruses. This is also the case for arthropod viruses in which DBV, one of the two known birnaviruses of drosophila, displays a very peculiar behavior.

Using X-ray structures of RdRp and capsid proteins to obtain reliable multiple sequence alignments and employing “up-to-date” phylogenetic methods, we revisited the pioneering work of Koonin and Dolja [45] in which they attempted to investigate the evolutionary relationships between +ssRNA and dsRNA virus families. This allowed us to obtain a more reliable picture of the evolutionary affinities between a number of viruses of the +ssRNA and dsRNA families.

Unraveling the +ssRNA and dsRNA virus evolutionary history is a complex undertaking since their genomes are subject to different rearrangements: recombination events between members of the same virus families, exchanges of segments for genomes with a multipartite organization, acquisition and loss of genes. Genome organizations for these viruses have been framed by both vertical and horizontal flows of genetic information. As suggested by Koonin and Dolja [46], they consist of a limited number of building blocks, the most universal ones being the set of genes coding for i) the RNA-dependent RNA polymerase that is the only protein conserved in all viruses and ii) the coat protein. Recently, the 3D structure determination of a number of virion architectures has revealed unsuspected similarities between the coat proteins of different viruses [53] that could further help in sorting out the relationship between viruses from different families.

## Abbreviations

RdRp: RNA-dependent RNA polymerase; ORF: Open reading frame; +ssRNA: Positive single strand RNA; dsRNA: Double-stranded RNA; BIC: Bayesian information criterion; KL divergence: Kullback-Leibler divergence; ML: Maximum likelihood; MCMC: Markov chain Monte Carlo; RB: Rotifer birnavirus; TV-1: Tellina virus; ESV: Esperito Santo virus; DAV: Drosophila A virus; DBV: Drosophila B virus; DXV: Drosophila X virus; IPNV: Infectious pancreatic virus; BSNV: blotched snakehead virus; IBDV: Infectious bursal disease virus.

## Competing interests

The authors declared that they have no competing interests.

## Authors’ contributions

JFG and MM performed the phylogenetic and structural analyses, BD carried out the RBV characterization, JFG, MM, PB and BD drafted the manuscript. JFG, MM, PB and BD participated in the design of the study. All authors read and approved the final manuscript.

## Supplementary Material

Additional file 1: Figure S1 Sequence alignment of birnavirus pVP2-specific domains. The alignment is anchored to the multiple cleavage sites (vertical arrows) experimentally identified on BSNV, IBDV, IPNV, TV-1 and DXV ([10] and references therein). Stars indicate residues conserved in the seven sequences. Colons or dots indicate conservative substitutions. Click here for file

Additional file 2: Figure S2 Birnavirus 7-taxon VP234 Bayesian consensus tree (MrBayes). Click here for file

Additional file 3: Figure S3 9-species ML tree with SH test results. Click here for file

Additional file 4: Figure S4 Tree-puzzle likelihood mapping. Tree-puzzle allows the user to evaluate the support of the internal branch of the three topologies generated by the four groups {Others, Flavi, Calici, Picorna}. The likelihood mapping diagram shows that 25% of the quartets cannot be resolved (i.e., they provide no information regarding any topology), 54% of the quartets favor the ML topology {Others, Flavi | Calici, Picorna}, 4% the topology {Others, Picorna | Calici, Flavi} (with 17% of the quartets providing some support to both topologies). No quartet favor the last topology {Others, Calici | Flavi, Picorna}. Click here for file

Additional file 5: Figure S5 12-species ML tree with SH test results. Click here for file

Additional file 6: Figure S6 13-species RdRp trees. Left: ML tree (PHYML); Right: Bayesian consensus tree (MrBayes). Click here for file

Additional file 7: Figure S7 Structural alignments of birna-, noda- and alphatetra-virus VP2 structures. Click here for file
