# Supplementary figures and images for: Analyses of the radiation of birnaviruses from diverse host phyla and of their evolutionary affinities with other double-stranded RNA and positive strand RNA viruses using robust structure-based multiple sequence alignments and advanced phylogenetic methods
Source: BMC Evol Biol. 2013 Jul 17;13:154. doi: 10.1186/1471-2148-13-154 (PMC3724706; doi:10.1186/1471-2148-13-154)

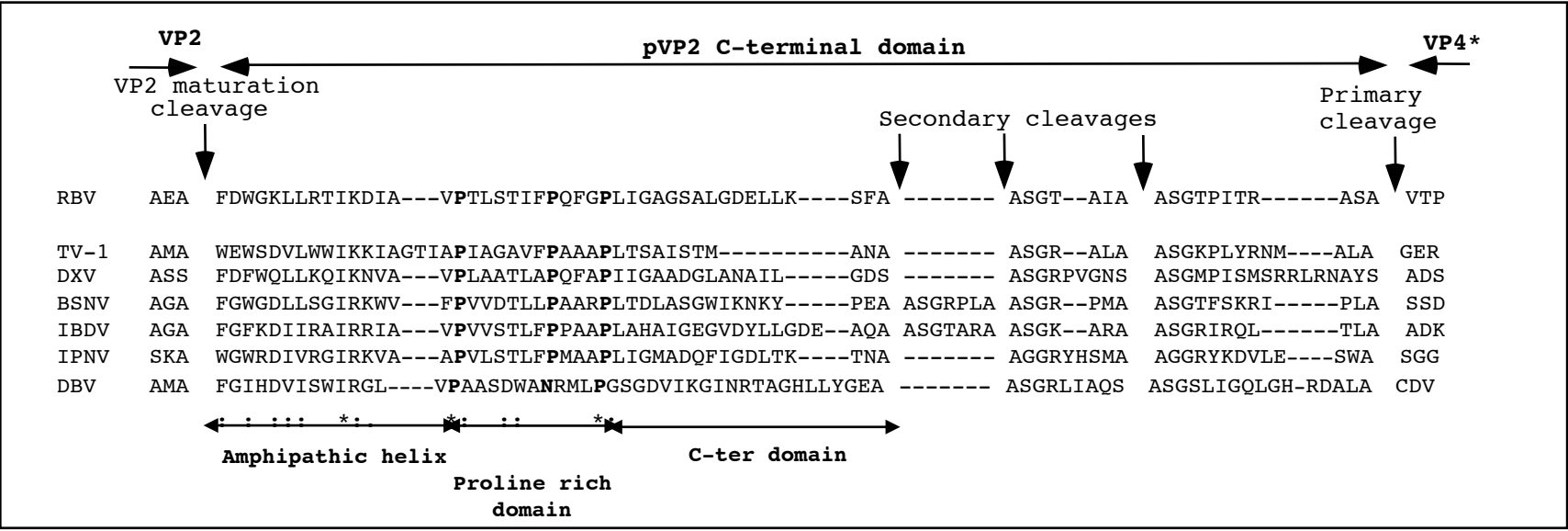

Supplement: Additional file 1: Figure S1 — Sequence alignment of birnavirus pVP2-specific domains. The alignment is anchored to the multiple cleavage sites (vertical arrows) experimentally identified on BSNV, IBDV, IPNV, TV-1 and DXV ([10] and references therein). Stars indicate residues conserved in the seven sequences. Colons or dots indicate conservative substitutions. [file 1471-2148-13-154-S1.pdf]

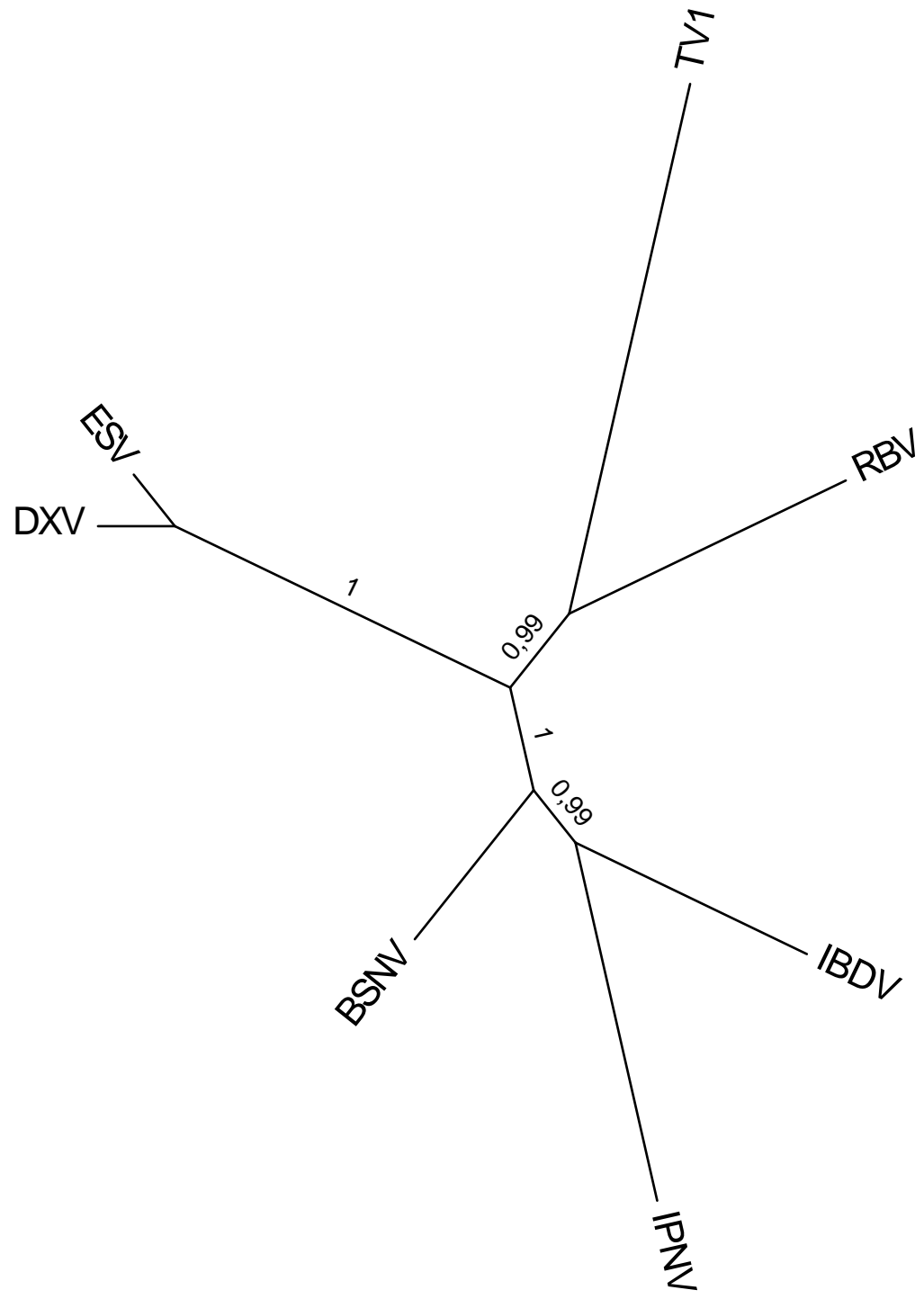

Supplement: Additional file 2: Figure S2 — Birnavirus 7-taxon VP234 Bayesian consensus tree (MrBayes). [file 1471-2148-13-154-S2.pdf]

RdRp9 ML tree with BP (EX\_EHO model)

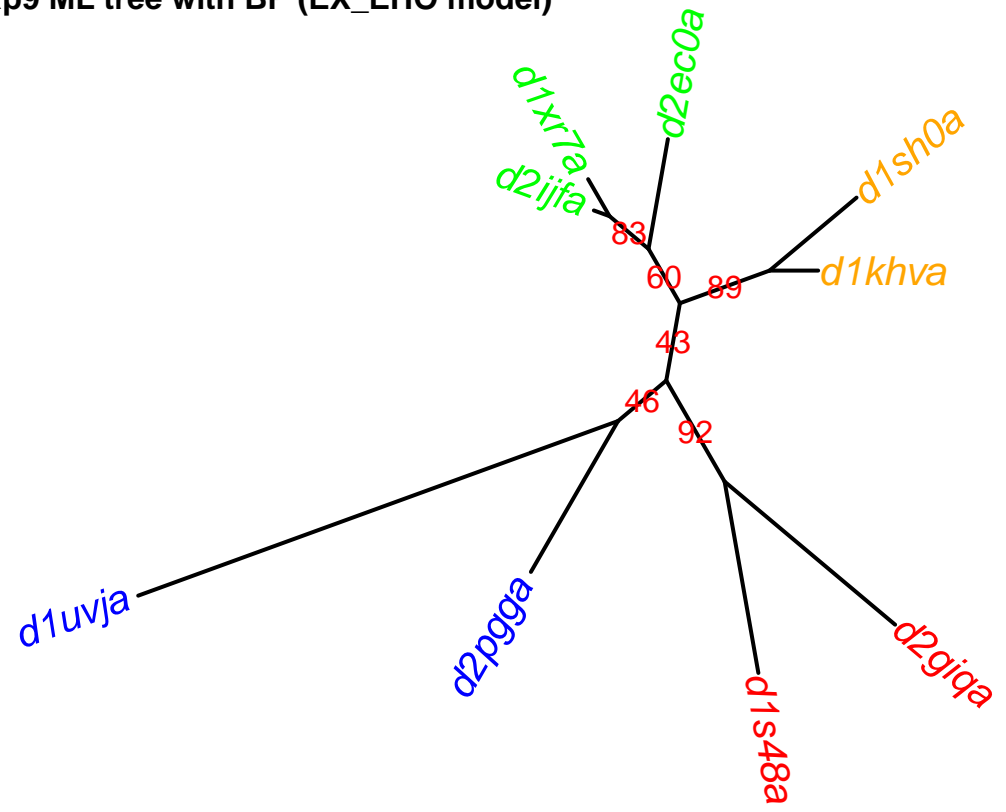

Topo. 2 (au: 0.743, np: 0.692)

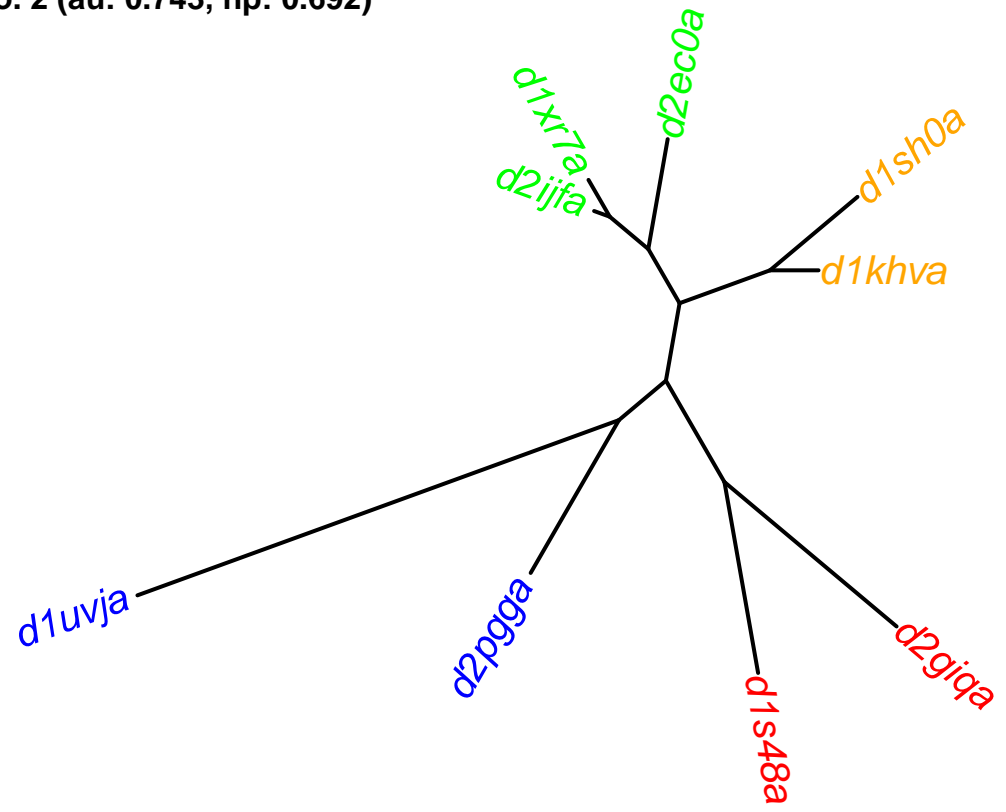

Topo. 1 (au: 0.325, np: 0.288)

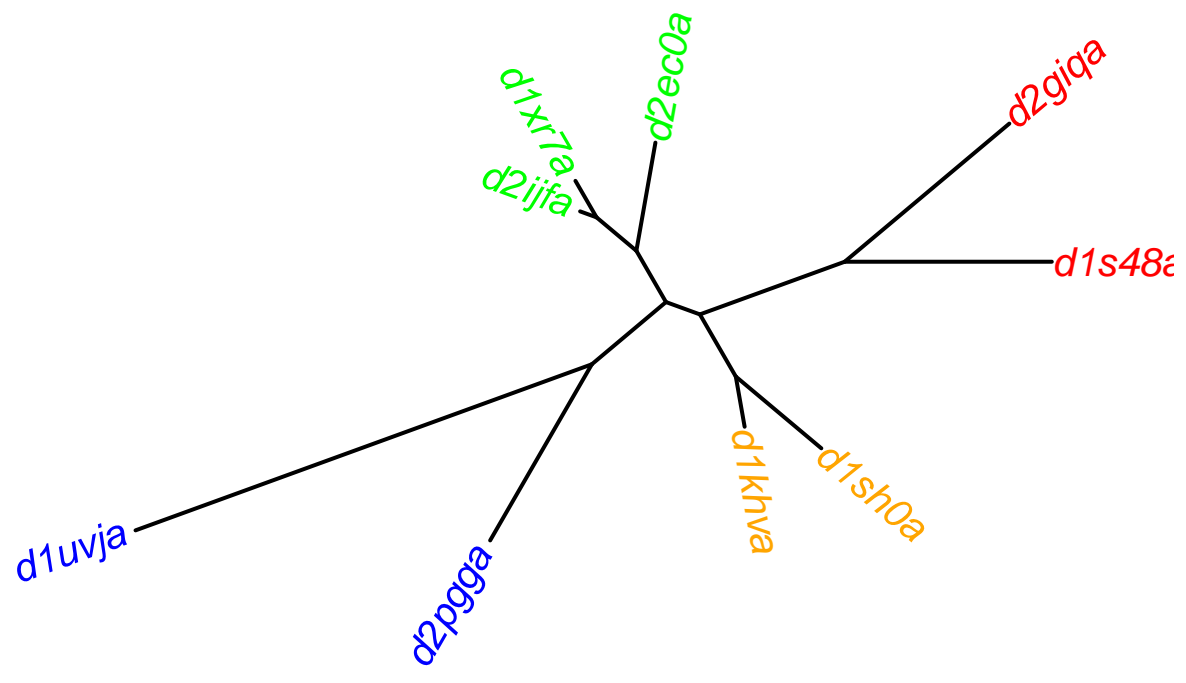

Topo. 3 (au: 0.065, np: 0.02)

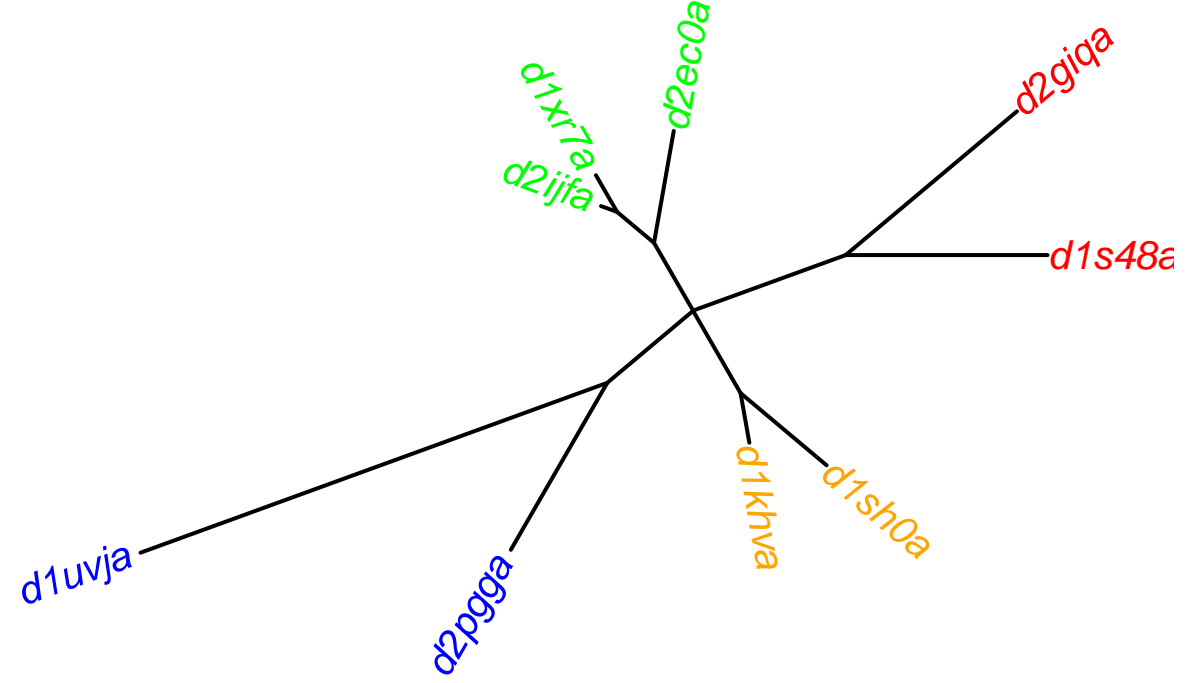

Supplement: Additional file 3: Figure S3 — 9-species ML tree with SH test results. [file 1471-2148-13-154-S3.pdf]

a = Others  
b = Flavi  
c = Picorna  
d = Calici

(a,b)-(c,d) max. likelihood tree

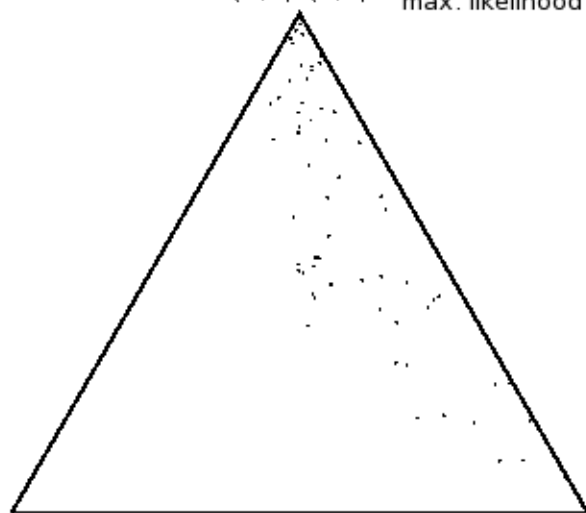

(a,d)-(b,c)

(a,c)-(b,d)

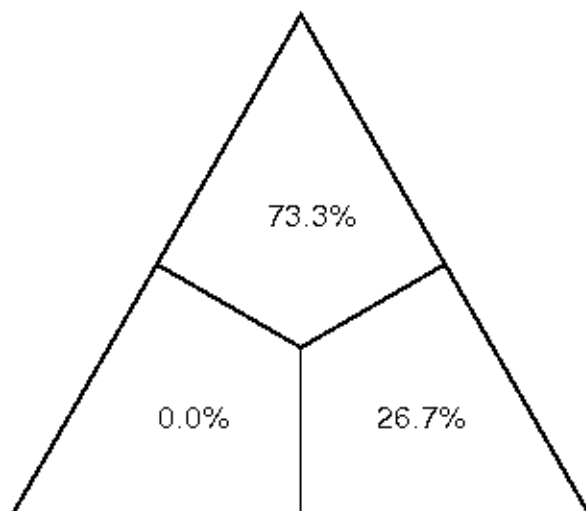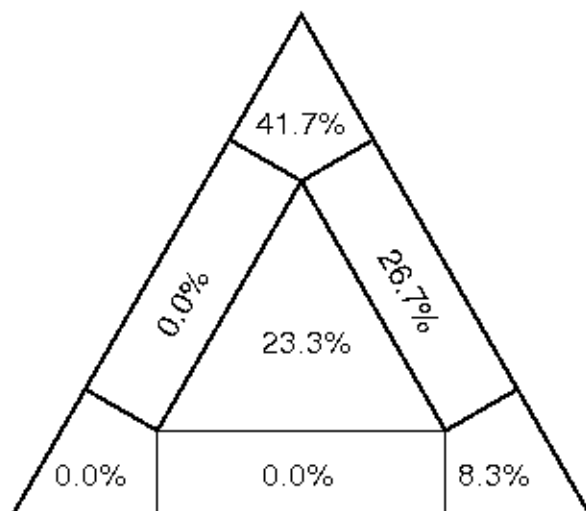

Supplement: Additional file 4: Figure S4 — Tree-puzzle likelihood mapping. Tree-puzzle allows the user to evaluate the support of the internal branch of the three topologies generated by the four groups {Others, Flavi, Calici, Picorna}. The likelihood mapping diagram shows that 25% of the quartets cannot be resolved (i.e., they provide no information regarding any topology), 54% of the quartets favor the ML topology {Others, Flavi | Calici, Picorna}, 4% the topology {Others, Picorna | Calici, Flavi} (with 17% of the quartets providing some support to both topologies). No quartet favor the last topology {Others, Calici | Flavi, Picorna}. [file 1471-2148-13-154-S4.pdf]

RdRp12 ML tree with BP (EX\_EHO model)

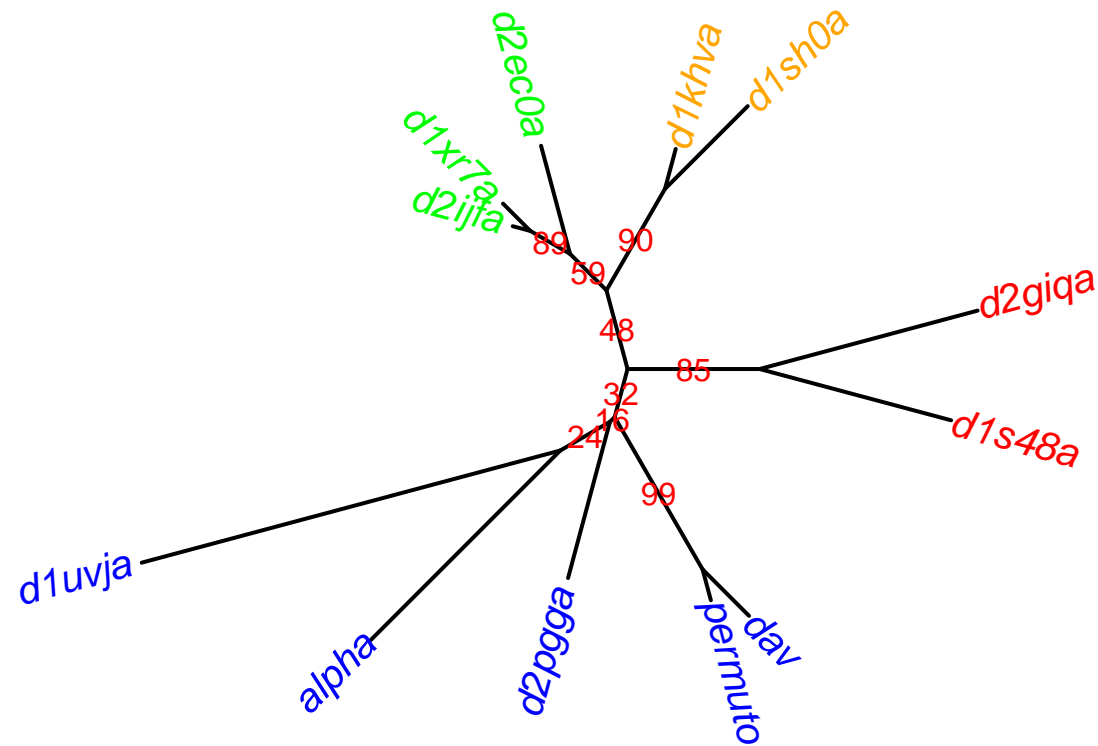

Topo. 2 (au: 0.775, np: 0.733)

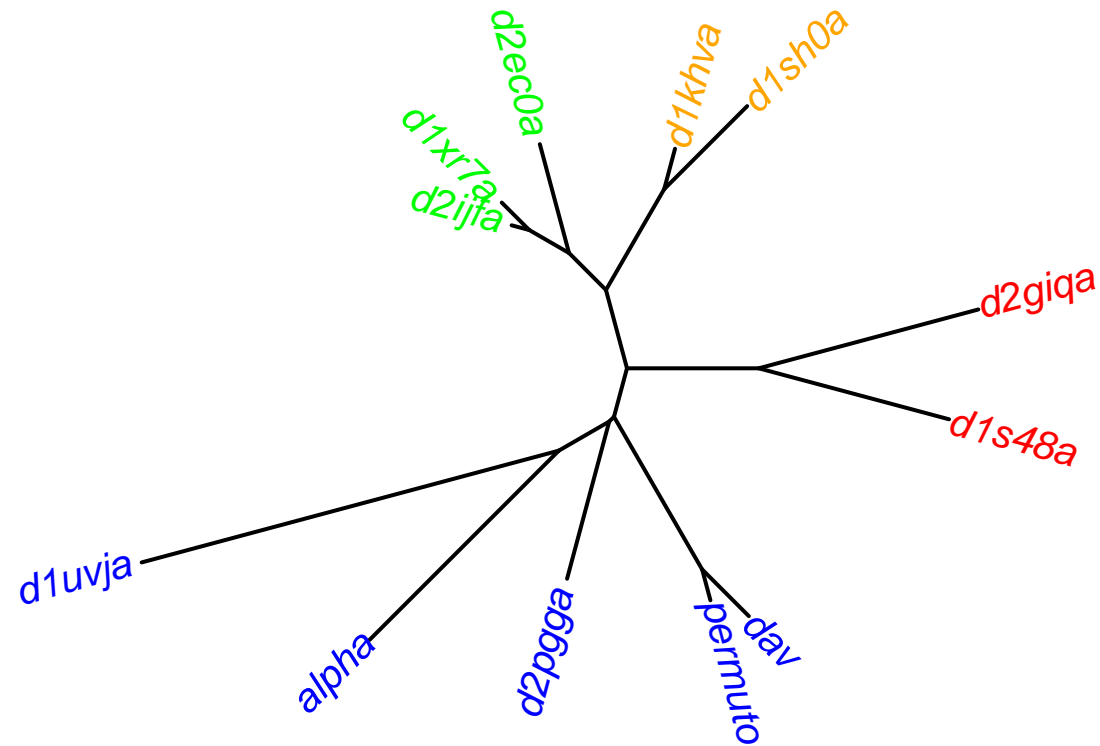

Topo. 1 (au: 0.284, np: 0.251)

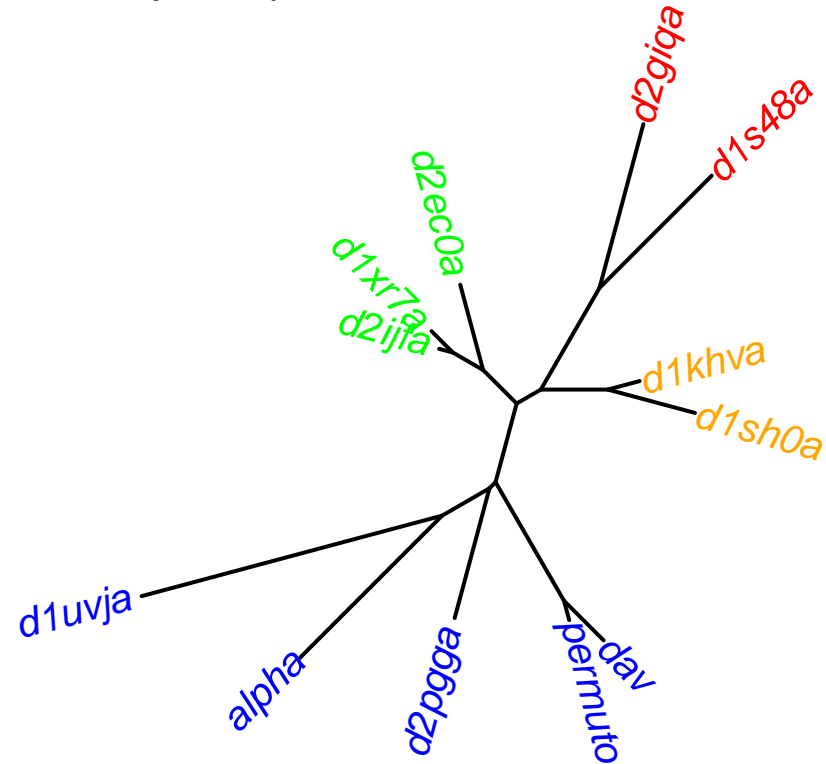

Topo. 3 (au: 0.052, np: 0.016)

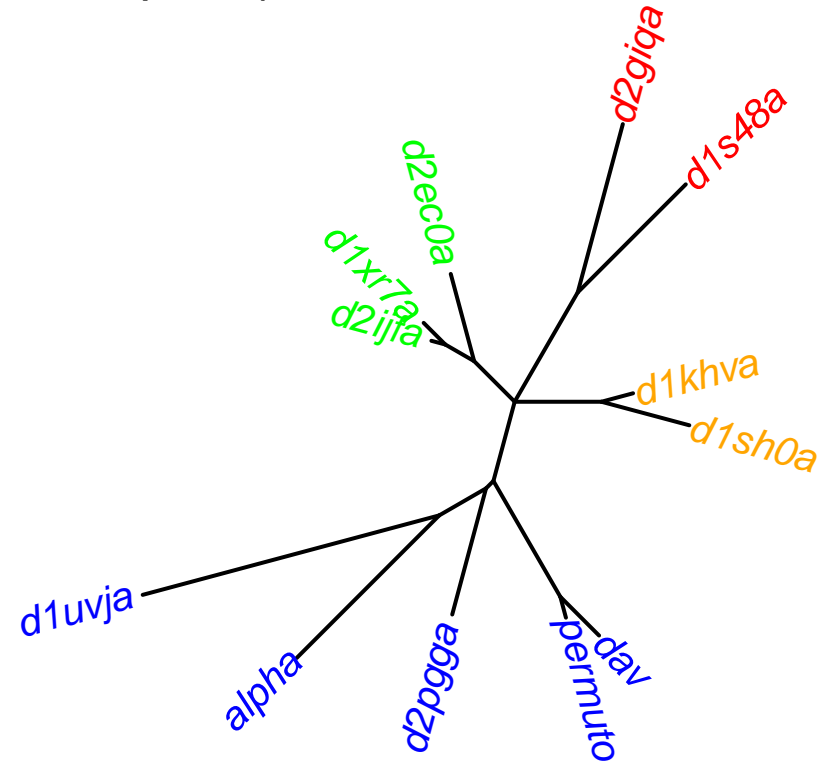

Supplement: Additional file 5: Figure S5 — 12-species ML tree with SH test results. [file 1471-2148-13-154-S5.pdf]

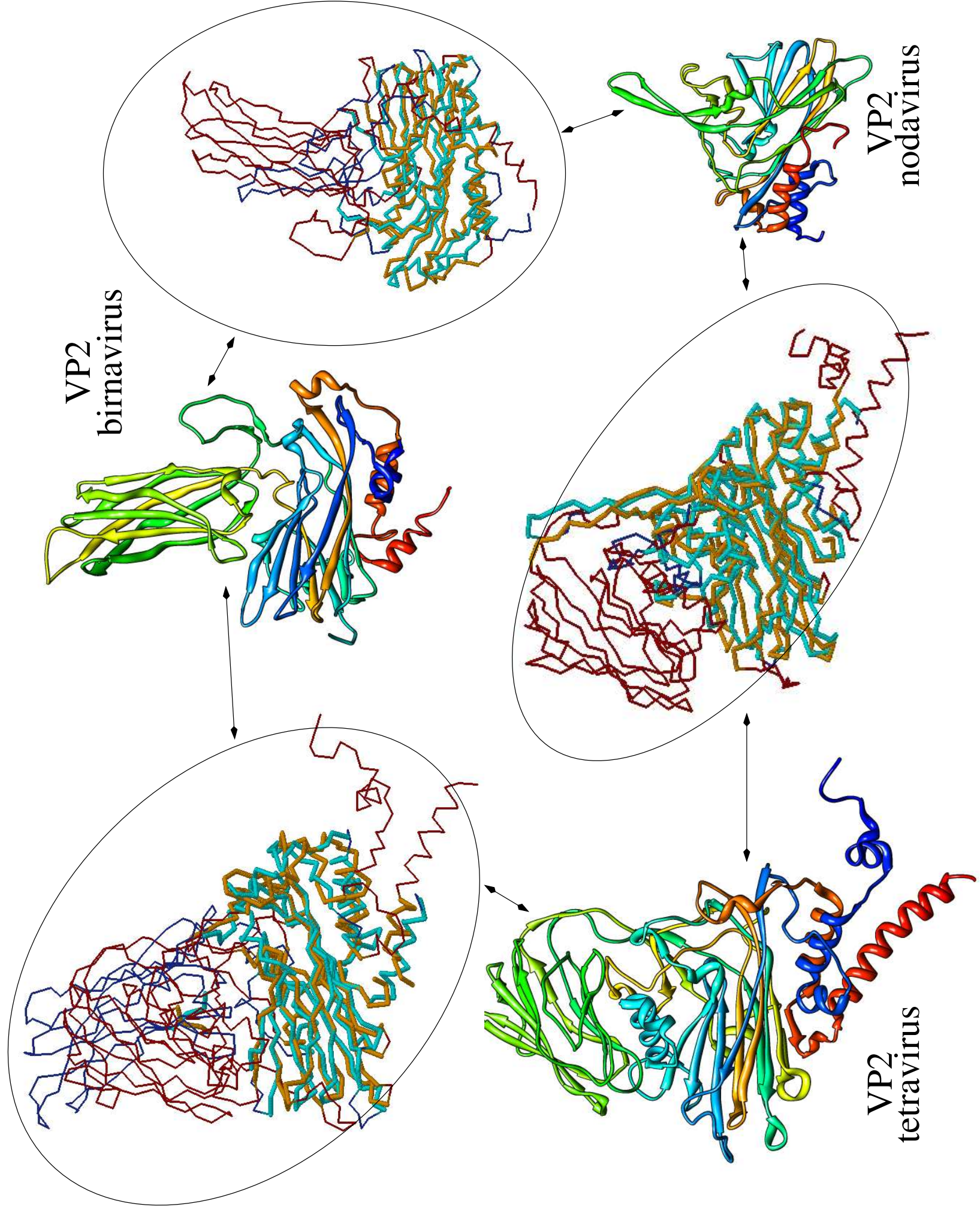

Supplement: Additional file 7: Figure S7 — Structural alignments of birna-, noda- and alphatetra-virus VP2 structures. [file 1471-2148-13-154-S7.pdf]
