# Supplementary material for: Analyses of the radiation of birnaviruses from diverse host phyla and of their evolutionary affinities with other double-stranded RNA and positive strand RNA viruses using robust structure-based multiple sequence alignments and advanced phylogenetic methods
Source: BMC Evol Biol. 2013 Jul 17;13:154. doi: 10.1186/1471-2148-13-154 (PMC3724706; doi:10.1186/1471-2148-13-154)
Supplement: Additional file 6: Figure S6 — 13-species RdRp trees. Left: ML tree (PHYML); Right: Bayesian consensus tree (MrBayes). [file 1471-2148-13-154-S6.pdf]

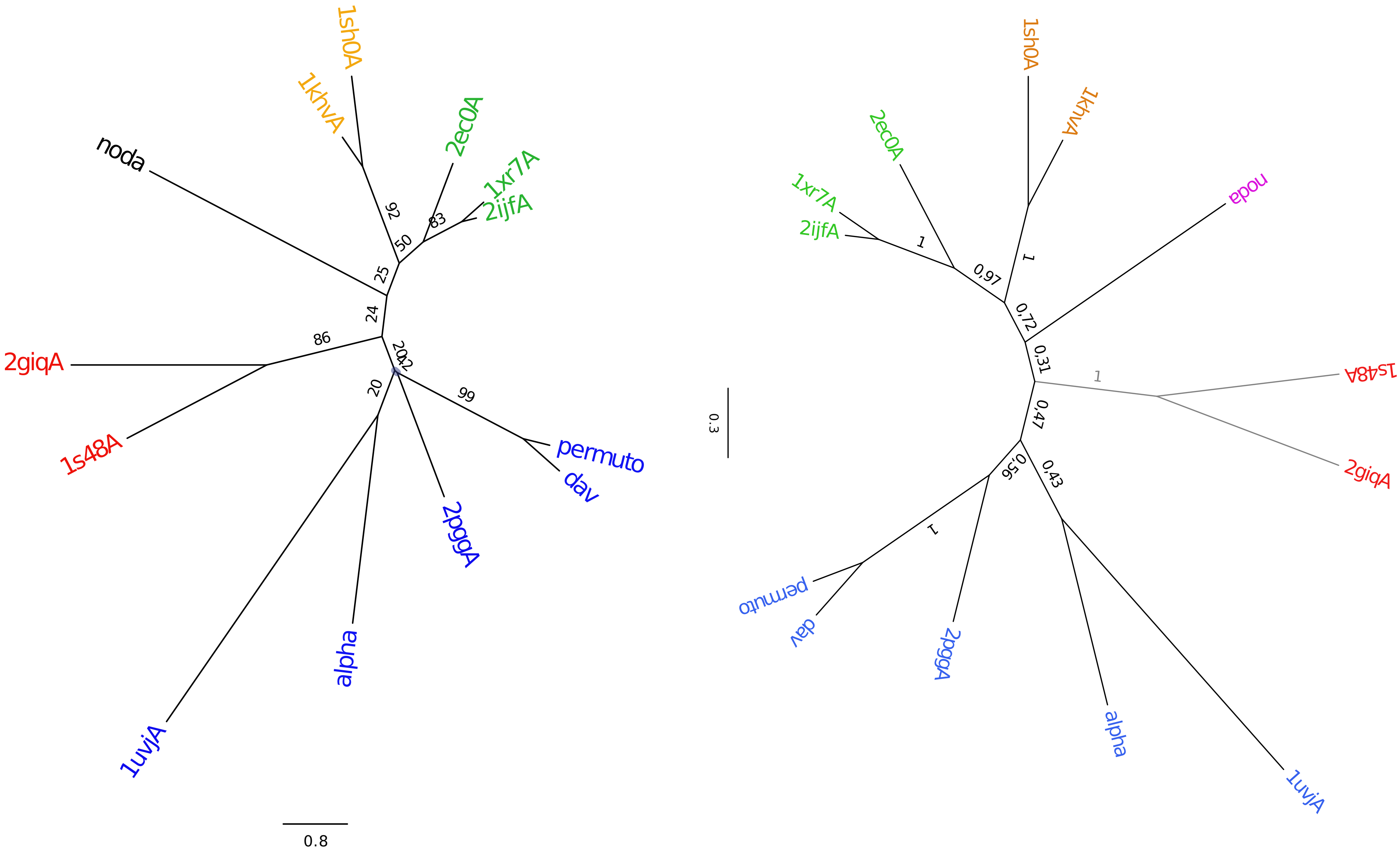

1sh0A
1khvA
2ec0A
1xr7A
2ijfA
noda
92
83
50
25
24
86
2giqA
1s48A
20
42
20
99
permuto
dav
2pggA
alpha
1uvjA
0.8
1sh0A
1khvA
2ec0A
1xr7A
2ijfA
noda
1
0,97
1
0,72
0,31
1
1s48A
2giqA
0,47
0,56
0,43
1
permuto
dav
2pggA
alpha
1uvjA
0.3
